# Modelling community-control strategies to protect hospital resources during an influenza pandemic in Ottawa, Canada

**DOI:** 10.1371/journal.pone.0179315

**Published:** 2017-06-14

**Authors:** Patrick Saunders-Hastings, Bryson Quinn Hayes, Robert Smith?, Daniel Krewski

**Affiliations:** 1 University of Ottawa, McLaughlin Centre for Population Health Risk Assessment, 850 Peter Morand Crescent, Ottawa, Ontario, Canada; 2 University of Ottawa, School of Epidemiology, Public Health, and Preventive Medicine, Faculty of Medicine, Ottawa, ON, Canada; 3 University of Ottawa, Department of Mathematics, Ottawa, ON, Canada; Georgia State University, UNITED STATES

## Abstract

**Background:**

A novel influenza virus has emerged to produce a global pandemic four times in the past one hundred years, resulting in millions of infections, hospitalizations and deaths. There is substantial uncertainty about when, where and how the next influenza pandemic will occur.

**Methods:**

We developed a novel mathematical model to chart the evolution of an influenza pandemic. We estimate the likely burden of future influenza pandemics through health and economic endpoints. An important component of this is the adequacy of existing hospital-resource capacity. Using a simulated population reflective of Ottawa, Canada, we model the potential impact of a future influenza pandemic under different combinations of pharmaceutical and non-pharmaceutical interventions.

**Results:**

There was substantial variation in projected pandemic impact and outcomes across intervention scenarios. In a population of 1.2 million, the illness attack rate ranged from 8.4% (all interventions) to 54.5% (no interventions); peak acute care hospital capacity ranged from 0.2% (all interventions) to 13.8% (no interventions); peak ICU capacity ranged from 1.1% (all interventions) to 90.2% (no interventions); and mortality ranged from 11 (all interventions) to 363 deaths (no interventions). Associated estimates of economic burden ranged from CAD $115 million to over $2 billion when extended mass school closure was implemented.

**Discussion:**

Children accounted for a disproportionate number of pandemic infections, particularly in household settings. Pharmaceutical interventions effectively reduced peak and total pandemic burden without affecting timing, while non-pharmaceutical measures delayed and attenuated pandemic wave progression. The timely implementation of a layered intervention bundle appeared likely to protect hospital resource adequacy in Ottawa. The adaptable nature of this model provides value in informing pandemic preparedness policy planning in situations of uncertainty, as scenarios can be updated in real time as more data become available. However—given the inherent uncertainties of model assumptions—results should be interpreted with caution.

## 1. Introduction

Influenza is an infectious disease that transmits between humans via inhalation of viral particles expelled by infected individuals during coughing or sneezing and carried in aerosol, respiratory droplets and fomites [1]. Though individuals experience infection differently, it often involves a combination of respiratory and systemic symptoms [2]. While generally self-limiting, influenza-related hospitalization and death is commonly associated with lower respiratory tract and neurological complications; in fact, influenza is the most deadly vaccine-preventable disease in North America [3]. As an RNA virus with a high mutation rate, humans are unable to maintain adequate long term immunity to influenza infection, leading to annual outbreaks of seasonal influenza [4]. In the United States, seasonal influenza accounts for between 3,000 and 49,000 deaths each year [5]. In recent years, the average number of influenza-associated hospitalizations and deaths in Canada is estimated to be approximately 12,000 and 3,500, respectively [6, 7].

These figures do not, however, reflect the more catastrophic potential of a pandemic influenza outbreak. Triggered by an antigenic shift—a reassortment of viral segments creating a new influenza strain—that creates a novel influenza virus to which humans possess little to no immunity, an influenza pandemic has the potential to transmit and spread rapidly across the globe [8]. This has happened on four occasions over the past one hundred years, leading to tens of millions of infections, hospitalizations and deaths [9–13]. While most of this burden was driven by the 1918 Spanish flu pandemic, even the most recent 2009 Swine flu pandemic is estimated to have resulted in as many as 575,400 deaths globally [12, 14], even though it is considered to be a mild strain compared to past pandemics [15]. Were a pandemic strain of a pathogenicity comparable to that of the 1918 outbreak to emerge today, projections suggest it could result in 21–31 million deaths worldwide [16].

Influenza pandemics emerge at uneven and unpredictable intervals [1, 17, 18]. The current global landscape, however, is one that is supportive of pandemic emergence. Unprecedented levels of human–animal interaction, viral diversity and extensive international travel collectively increase the threat of viral reassortment, crossover to humans and global spread [19–24]. The threat of a global pandemic and its potential consequences present major challenges for public health and emergency preparedness policy efforts.

Given that the epidemiological features of a pandemic cannot be known until after its emergence, there is significant uncertainty regarding best practices in resource- and control-strategy planning [25, 26]. This is problematic, as pandemic planning is crucial to limiting illness, death and essential-service disruption, thereby mitigating the health, social and economic burden of pandemics. Mathematical models of disease transmission, accounting for the uncertainty and randomness inherent in pandemic emergence and spread, have become valuable tools in pandemic planning and management [27, 28]. Given that empirical field studies in pandemic situations are generally infeasible or unethical, they are of vital importance. Unfortunately, important gaps persist in the field of pandemic modelling, particularly with respect to accessibility, assessment of resource capacity and economic evaluation [29]. The model presented herein seeks to address these gaps.

This paper presents initial findings generated by InFluNet, a new, discrete-time simulation model that combines ordinary differential equations (ODEs) with stochastic approaches. Designed to capture the range of factors influencing pandemic transmission—while remaining accessible to public-health practitioners lacking formal modelling training—InFluNet combines epidemiological and demographic data to allow prediction of the health and economic burdens associated with future pandemics. Emphasizing the adequacy of hospital-resource capacity, the model allows evaluation of the potential for community intervention strategies to contain pandemic spread and ensure hospital resource adequacy. Results should inform policy-planning for interventions targeting specific stages of the disease life-cycle, and specific settings (household, school, workplace, and community), supporting more efficient and cost-effective control strategies.

## 2. Methods

InFluNet is a population-level, discrete-time simulation model that builds on previous deterministic and stochastic influenza models [30–37]. The model combines differential equations estimating the rate of transmission contacts and disease progression with stochastic methods of estimating social-mixing behaviour and transmission probability. This dual approach effectively describes the average behaviour of larger urban populations, while incorporating the uncertainty associated with transmissibility and pathogenicity of a new influenza strain. This section describes the InFluNet simulation model, as well as the data inputs and outputs associated with the model. No ethical approval was required for this study, as all hospital data analyzed were previously anonymized and publically available. No individual patient data were independently analyzed in this research. The model is described in brief below, with a more detailed discussion of the model structure, background and approach included in **S1 File**.

### 2.1 Social contact network

The model is structured to represent three independent transmission–time/location steps over the course of the day: household, school/work and community. These estimates are based upon empirical data from a representative North American municipality [38] and closely resemble contact rate assumptions from past influenza modelling studies of Ontario cities [33, 39]. InFluNet uses ODEs to estimate age-specific contact rates between various age groups. Five age groups are identified: infant (0–4), child (5–18), young adult (19–29), adult (30–64) and senior (65+). This reflects age groupings of past studies [30, 36, 37, 40], based on previously observed patterns of pandemic influenza transmission and outcomes. Individuals will interact with others at different rates, both within and outside their age groups; daily contact rates by age and location are included in **S1 File (S1.1)**.

### 2.2 Transmissibility

InFluNet uses a “next-generation operator” approach described previously in a model of smallpox [41] and reflective of a heterogeneous population [42]. In this approach, transmissibility can be described as follows:

$$\beta =\beta_{C}+\beta_{A}+\beta_{AV}$$ (1)

where

$$\beta_{C}=\gamma \cdot \alpha_{C}\cdot \eta_{C}\cdot \left(1-e^{-\sigma \tau }\right)\cdot \frac{I_{C}}{N}$$ (2)

$$\beta_{A}=\gamma \cdot \alpha_{A}\cdot \eta_{A}\cdot \left(1-e^{-\sigma \tau }\right)\cdot \frac{I_{A}}{N}$$ (3)

and

$$\beta_{AV}=\gamma \cdot \alpha_{AV}\cdot \eta_{AV}\cdot (1-e^{-\sigma \tau })\cdot \frac{I_{AV}}{N}$$ (4)

The rate of disease transmission from symptomatic (β_C_), asymptomatic (β_A_) and symptomatic treated (β_AV_) individuals depends on the six parameters [38, 41] described in **Table 1**. More information on our transmissibility assumptions is available in **S1 File (S1.2)**.

**Table 1:** Transmissibility function parameters.

Symbol	Definition	Sample value	References	Range
γ	Number of effective contacts	As per contact tables	[41]	0.01–10 (contacts/day)
α	Susceptibility	1.0 for infants, children, and young adults; 0.95 for adults; 0.65 for seniors	[43]	0–1
η	Infectivity	1.0	Assumed	0–1
σ	Duration of contacts	As per contact tables	[41]	1/2–1/6 (days/contact)
τ	Mean number of infections per time within a contact between a susceptible and infected individual, assuming full infectivity and susceptibility	0.275	[16, 26, 44, 45]	0.17–0.42

### 2.3 Model structure

**Fig 1:**
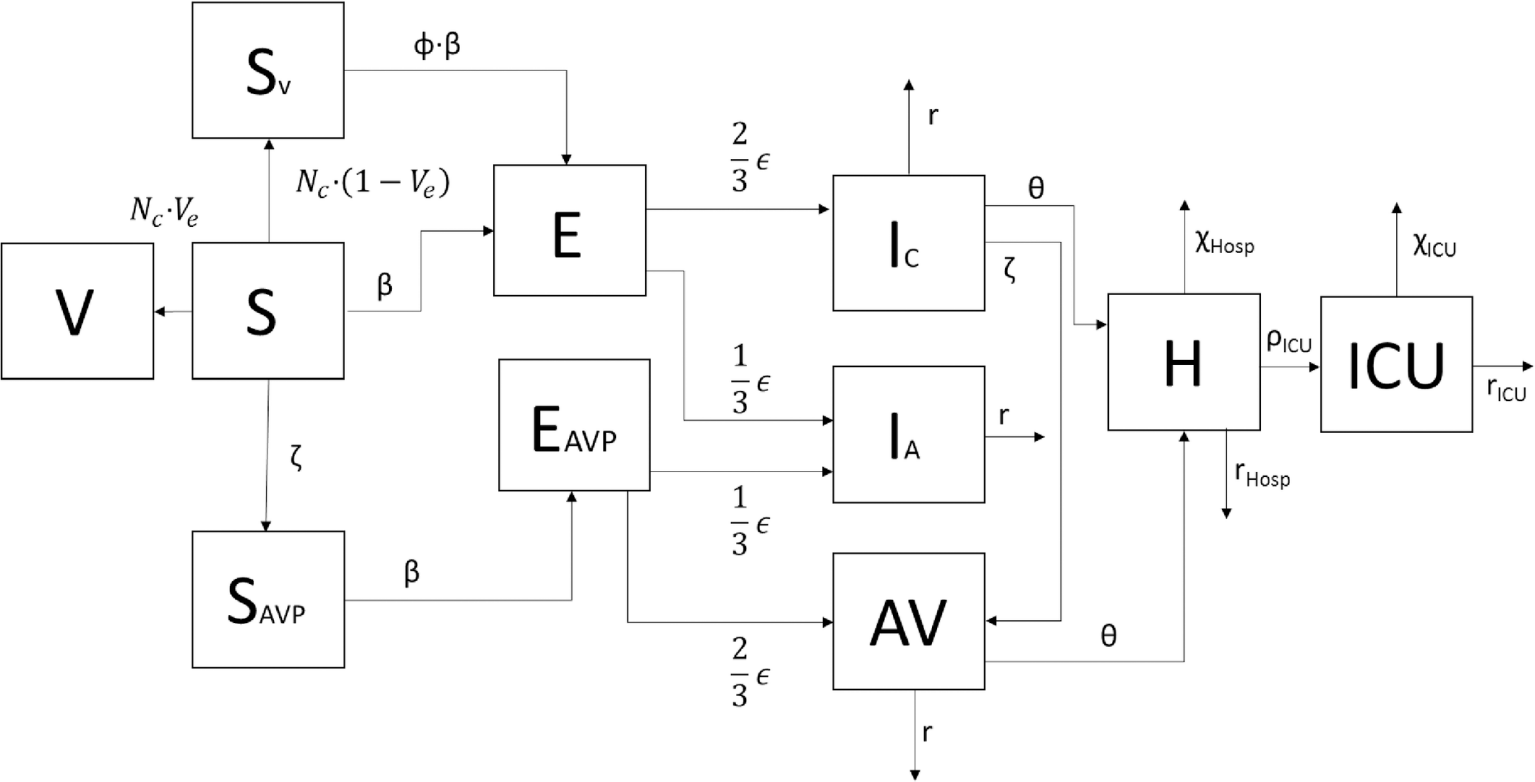
Transmission model flow diagram.

InFluNet combines demographic, hospital and intervention data, allowing simulation of a pandemic influenza outbreak under millions of possible scenarios. A simulation is run five times, with the output value being averaged across simulations and reported with 95% confidence intervals calculated via the standard-deviation approach. The InFluNet model follows the general structure of an age-dependent SEIR (susceptible-exposed-infected-recovered) model, a structure that has predicted outbreaks with relative accuracy in the past [46–49]. The transmission model flow diagram is illustrated in **Fig 1**.

Susceptible individuals (S) can receive successful vaccination (V), failed vaccination (S_V_) or antiviral prophylaxis (AVP). While individuals in the “V” group are protected, individuals in “S”, “S_V_” or “AVP” compartments can be infected, moving into a latent period (E). From here they become either symptomatically (I_C_) or asymptomatically (I_A_) infectious. Of those in the “I_C_” group, some will require antiviral therapy (AVT), hospitalization (H) and ICU care (ICU). A small proportion of those in the “I_C_”, “H”, and “ICU” groups will experience a fatal infection. From the flow chart in **Fig 1**, we arrive at the system of ODEs presented below. A detailed discussion of the model structure and parameters is included in **S1 File (S1.3)**. Intervention parameters are discussed in **Section 2.4**.

$$\frac{dS}{dt}=-(N_{C}+\zeta +\beta )\cdot S\;\mathrm{Susceptible}$$ (5)

$$\frac{dE}{dt}=\beta \cdot \left(S+\phi \cdot S_{V}\right)-E\cdot \epsilon \;\mathrm{Latent}\;\mathrm{Infected}$$ (6)

$$\frac{dS_{V}}{dt}=(1-V_{e})\cdot N_{C}\cdot S-\phi \cdot S_{V}\cdot \beta \mathrm{Susceptible}\;\mathrm{with}\;\mathrm{failed}\;\mathrm{vaccination}$$ (7)

$$\frac{dV}{dt}=V_{e}\cdot N_{C}\cdot S\;\mathrm{Vaccinated}$$ (8)

$$\frac{dS_{AVP}}{dt}=\zeta \cdot S-\beta \cdot S_{AVP}\;\mathrm{Antiviral}\;\mathrm{prophylaxis}$$ (9)

$$\frac{dE_{AVP}}{dt}=\beta \cdot S_{AVP}-E_{AVP}\cdot \epsilon \;\mathrm{Latent}\;\mathrm{infected}\;\mathrm{treated}\;\mathrm{with}\;\mathrm{prophylaxis}$$ (10)

$$\frac{dI_{C}}{dt}=\frac{2}{3}\cdot E\cdot \epsilon -I_{C}(\theta +r+\zeta )\;\mathrm{Infected}\;\mathrm{Symptomatic}$$ (11)

$$\frac{dI_{A}}{dt}=\frac{1}{3}\cdot \left(E+E_{AVP}\right)\cdot \epsilon -I_{A}\cdot r\;\mathrm{Infected}\;\mathrm{Asymptomatic}$$ (12)

$$\frac{dAV}{dt}=I_{C}\cdot \zeta +\frac{2}{3}\cdot E_{AVP}\cdot \epsilon -AV(\theta +r)\;\mathrm{Treated}\;\mathrm{with}\;\mathrm{Antivirals}$$ (13)

$$\frac{dH}{dt}=\left(I_{C}+AV\right)\cdot \theta -\left(r_{Hosp}+\chi_{Hosp}+\rho_{ICU}\right)\cdot H\;\mathrm{Hospitalized}$$ (14)

$$\frac{dICU}{dt}=\rho_{ICU}\cdot H-\left(r_{ICU}+\chi_{ICU}\right)\cdot ICU\;\mathrm{Intensive}\;\mathrm{Care}\;\mathrm{Unit}$$ (15)

Patient-demand data is plotted against hospital-resource data obtained from the Canadian Institute for Health Information and Québec Ministry of Health and Social Services [50, 51]. Combining these hospital-resource data with the Census demographic data, we generated individual profiles for each Canadian CMA; details on hospital-resource estimation are included in **S1 File (S1.4)**, while the Ottawa–Gatineau profile used for the following analysis is included in **S1 Table**.

The model generates results relating to health and economic outcomes. Health outcomes summarize consequences in terms of the number of symptomatic cases, hospitalizations, ICU admissions, ventilator demand and deaths by age, location and as a percentage of existing capacity. These data are reported on a daily basis and summarized over the course of the entire outbreak. Economic evaluations are conducted in two ways: by estimating the overall economic burden associated with a pandemic by aggregating influenza- and intervention-associated costs, and by calculating the relative cost-effectiveness of different intervention strategies. The latter is assessed by calculating the cost per life-year saved, relative to a “no intervention” scenario. Economic estimates are generated by scaling health and intervention endpoints (as generated by the model) by a per-case economic cost, as given in **Table 2**. The internationally proposed cost-effectiveness threshold of CAD $50,000 and a 1.5% annual discounting rate were used to calculate costs associated with mortality [52–55]. A more detailed discussion of model outputs is included in **S1 File (S1.5)**.

**Table 2:** Economic cost ($CAD) for outcomes of interest.

Economic consequences		
Category	Unit Cost	Citation
Total hospital bed days	$1,042/day	[56, 57]
Total ICU + ventilator days	$2,084/day	[56]
Total deaths	0–4: $2,355,172 5–18: $2,207,744 19–29: $1,956,694 30–64: $1,374,086 65+: $424,296	[52, 53]
Total lost school days	$91.85/day	[33, 58]
Total lost work days	$192.55/day	[59]
Total vaccinations	$20.00/vaccination	[56]
Total courses of antivirals	$25.00	[56, 57, 60]
Total masks	$4.00/mask	Estimated

### 2.4 Intervention effectiveness

InFluNet allows the evaluation of eight different interventions (vaccination, antiviral treatment, antiviral prophylaxis, school closure, community-contact reduction, personal protective measures, voluntary isolation and quarantine), with coverage, intensity and effectiveness measures determined by user inputs. In this study, we provide a “Best Guess” (BG), “Worst Case” (WC), and “Best Case” (BC) for each parameter, in order to generate a range of estimated intervention impacts. **Tables 3** and **4** summarize the parameter definitions and user input ranges for each intervention parameter. The tables also gives the parameter values we used in this modelling study, as well as the sources that informed these values. Where possible, empirical values from the 2009 pandemic were used; where these were unavailable, we used assumptions employed in past modelling studies. Each intervention is also discussed in detail in **S1 File (S1.6)**.

**Table 3:** Pharmaceutical intervention parameters. Best-guess scenarios reflect pooled estimates as available. Worst- and best-case scenarios (in terms of disease transmission but not necessarily resource allocation) reflect 95% confidence intervals where available; otherwise, they reflect ranges of estimates reported.

Intervention	Parameter^τ^	Theoretical Range	Worst Case Scenario	Best Guess	Best Case Scenario	Citation
Pandemic vaccination	Time delay (weeks)	0–4	4	0	Pre-vaccination	[16, 31]
	Coverage (%)*	0–100	25	35	45	[56, 61]
	Effectiveness (susceptibility) (%) */**	0–100	40	65	90	[40, 62–65]
	Effectiveness (infectivity) (%)**	0–100	20	35	50	[40, 65]
	Reduction in hospitalization rate (%)*	0–100	25	60	90	[66]
Antiviral treatment	Coverage (% infected that seek treatment)**	0–100	30	50	70	[67]
	Effectiveness (infectivity) (%)*	0–100	57	75	82	[31, 39, 68–70]
	Reduction in hospitalization rate (%)*	0–100	0	10	40	[71]
	Resistant strain	Yes/No	Yes	No	No	None
Antiviral prophylaxis	Coverage (% of households, workplaces, and schools receiving prophylaxis)**	0–100	10	35	60	[30]
	Effectiveness (susceptibility) (%)*	0–100	10	30	50	[32, 71]
	Effectiveness (infectivity) (%)*/**	0–100	57	75	82	[31, 39, 68–70]
	Reduction in hospitalization rate (%)*	0–100	0	10	40	[71]
	Resistant strain	Yes/No	Yes	No	No	None

^τ ^* approximated from empirical studies

** approximated from modelling studies

*** approximated from qualitative studies

**Table 4:** Non-pharmaceutical intervention parameters. Best-guess scenarios reflect pooled estimates as available. Worst- and best-case scenarios (in terms of disease transmission but not necessarily resource allocation) reflect 95% confidence intervals where available; otherwise they reflect ranges of estimates reported.

Intervention	Parameter^τ^	Theoretical Range	Worst Case Scenario	Best Guess	Best Case Scenario	Citation
School closure	Adults that will be redistributed (%)*	0–100	8	20	33	[72]
Community-contact reduction	Reduction in community-contact rate (%)**	0–100	25	50	75	[31, 60, 73, 74]
Hand hygiene	Effectiveness (%)*	0–100	3	26	44	[75–79]
	Adherence (%)*	0–100	20	38	55	[75, 80–82]
Mask use	Effectiveness (%)*	0–100	8	60	82	[83–87]
	Adherence (%)*	0–100	1	3	5	[88, 89]
Voluntary isolation	Adherence (%)**	0–100	10	30	50	[90, 91]
Quarantine (subtracted from VI adherence	Adherence (%)***	0–100	5	15	25	[92]

^τ:^ * approximated from empirical studies

** approximated from modelling studies

*** approximated from qualitative studies

### 2.5 Analysis

We present summary values for six key outcome measures—cases of illness, hospitalization, ICU admission, peak acute care demand as a percentage of capacity, peak ICU demand as a percentage of capacity and death—across all 192 simulations conducted. Multivariate sensitivity analyses were conducted to evaluate how changes in disease and intervention characteristics might affect the evolution, progression and control of the pandemic. The effect of disease parameters was evaluated by conducting simulations under different transmissibility and pathogenicity assumptions. The effect of intervention parameters was evaluated by comparing BC and WC intervention scenarios to the BG scenario.

We also present the results of a complete analysis for seven key intervention bundles of interest. The scenarios and reasons for their emphasis are indicated below. Taken together, this two-tiered analysis should provide the breadth and depth of analysis needed to inform future pandemic planning.

1. No intervention: establish a baseline prediction.
2. Vaccination and antiviral treatment: assess the impact of commonly implemented pharmaceutical interventions in the absence of any non-pharmaceutical measures.
3. Vaccination, antiviral treatment and antiviral prophylaxis: assess the impact of the full range of pharmaceutical interventions that could be employed.
4. Community-contact reduction, personal protective measures and voluntary isolation: assess the impact of minimally invasive non-pharmaceutical interventions in cases where pharmaceutical measures may be unavailable.
5. School closure, community-contact reduction, personal protective measures, voluntary isolation and quarantine: assess the impact of the full scope of non-pharmaceutical measures in cases where pharmaceutical measures may be unavailable.
6. Community-contact reduction, personal protective measures, voluntary isolation and antiviral treatment: assess the impact of minimally disruptive non-pharmaceutical measures and antiviral treatment in cases where vaccination may not yet be available.
7. All interventions: assess the impact of implementing the full range of available interventions.

### 2.6 Validation

The InFluNet model was validated using four approaches, as informed by a recent review of mathematical modelling validation protocols [29]: parameterization, sensitivity analysis, structural validation and predictive validation. Parameterization involves the selection of values from empirical data; this was done wherever possible, prioritizing Canadian contexts where data were available. Multivariate sensitivity analyses were conducted to evaluate the impact of change in certain disease and intervention parameters, as described in Section 2.5. Structural validity refers to the extent to which the model is consistent with current theory and practices, reflecting the way in which the real-world system works: InFluNet is based in epidemic theory and builds on the work of previously published transmission [38, 41] and intervention [33, 56, 93, 94] modelling research.

Predictive validation assesses the extent to which the model will produce accurate data: this is the most difficult element of mathematical modelling validation, particularly in the case of pandemic influenza, where there is such heterogeneity in the disease, population and intervention parameters, and the majority of disease transmission processes are unreported and invisible to health-surveillance agencies. In an effort to overcome this challenge, we fit the model to past experiences of the 2009 pandemic by estimating values for the transmissibility parameter τ, which was estimated numerically from initial conditions and contact rates [38, 41]. We then conducted an assessment of the model’s predictive validity by comparing its predicted attack rate in a pandemic scenario with a transmissibility parameter representative of the 2009 H1N1 pandemic to both empirical data [25, 95] and the results of a previously published pandemic study that calibrated its inputs to the 2009 H1N1 pandemic [33]. Taking Hamilton, Ontario, as a simulated study population, Andradottir *et al* [33] constructed a simulation model that predicted an illness attack rate of 36.8%; taking the Ottawa–Gatineau CMA as a study population under similar assumptions, InFluNet predicted an illness attack rate of 41.0% (95% CI: 40.9–41.2%), though it was noted that Andradottir *et al* assumed a higher rate of pre-existing immunity. Given this, the small differences were interpreted as supportive of the predictive validity of our model. In this way, we validated our transmissibility parameter estimates against both the basic reproduction number of previous models [33] and empirical Canadian pandemic H1N1 attack rates [25, 95]. This was essential to anchoring a representative transmissibility parameter, as it is not enough to assume that reproductive rates will be identical across differential equation models with different transmission assumptions.

## 3. Results

The subsections below present results related to symptomatic influenza infection, hospitalization, ICU admission, hospital resource demand, mortality and economic burden. **Table 5** summarizes the basic findings of the seven key intervention scenarios of interest. Results from all 192 simulations are discussed in their respective subsections, with summary tables included in the supplementary material.

**Table 5:** Summary of health-outcome measures from simulations of seven key intervention scenarios.

Intervention*	Outcome			
	Symptomatic Cases	Hospitalizations	ICU	Deaths
**None**	677,546	2,472	580	363
**V+AVT**	622,681	815	192	118
**V+AVT+AVP**	600,394	765	180	109
**CCR+PPM+VI**	203,771	634	151	65
**CCR+PPM+VI+AVT**	200,537	560	133	58
**SC+CCR+PPM+Q**	189,015	550	132	56
**SC+CCR+PPM+Q+ V+AVT+AVP**	104,051	108	26	11

*V = vaccination; AVT = antiviral treatment; AVP = antiviral prophylaxis; CCR = community-contact reduction; PPM = personal protective measures; VI = voluntary isolation; Q = voluntary isolation and quarantine; SC = school closure

### 3.1 Symptomatic infection

Under assumptions reflective of an influenza pandemic of transmissibility similar to the 1957 H2N2 pandemic, the InFluNet model projected that, in the absence of any intervention, about 677,546 symptomatic influenza infections would occur in the Ottawa–Gatineau CMA. This amounts to an illness attack rate of 53.4%. No single intervention implemented in isolation successfully brought the attack rate under 30%. Of the eight interventions, vaccination, personal protective measures, combined voluntary isolation and quarantine procedures resulted in the greatest reductions, producing attack rates of 50.0%, 45.5% and 33.9%, respectively. Antiviral treatment, antiviral prophylaxis, school closure and community-contact reduction produced only small reductions in illness attack rate, whether implemented alone or in combination with other interventions.

The timely initiation of multiple pandemic control measures resulted in significant reductions in symptomatic case numbers. This was particularly true when vaccination was combined with personal protective measures and isolation of infected individuals. Even in the absence of any pharmaceutical intervention, adherence to rigorous non-pharmaceutical protocols—school closure, community-contact reduction, personal protective measures, voluntary isolation and quarantine—resulted in a reduction of the illness attack rate to 15.2%, by delaying peak pandemic transmission beyond the 100-day simulation interval. Modelling of all eight interventions implemented in conjunction reduced the illness attack rate to 8.4%. Results of all 192 simulations are included in **S2 Table**.

With no intervention, the relative proportions of symptomatic infection approximately mirrored the age-stratified population distribution. **Table 6** presents cases of symptomatic infection by age group, along with a calculation of the percentage of all cases represented by each age group.

**Table 6:** Predicted number of cases of symptomatic infection by intervention type, and percentage of total infections accounted for by each group.

Intervention	Age group					
	Infant	Child	Young adult	Adult	Senior	Total
**None**	39,628	111,066	103,976	344,858	78,018	677,546
	5.8%	16.4%	15.3%	50.9%	11.5%	100.0%
**V+AVT**	36508	101985	95567	317617	71004	622681
	5.9%	16.4%	15.3%	51.0%	11.4%	100.0%
**V+AVT+AVP**	35270	98279	92158	306757	67930	600394
	5.9%	16.4%	15.3%	51.1%	11.3%	100.0%
**CCR+PPM+VI**	17427	36467	24842	105413	19622	203771
	8.6%	17.9%	12.2%	51.7%	9.6%	100.0%
**CCR+PPM+VI+AVT**	17183	35893	24427	103747	19287	200537
	8.6%	17.9%	12.2%	51.7%	9.6%	100.0%
**SC+CCR+PPM+Q**	17998	53368	25040	72406	20204	189016
	9.5%	28.2%	13.2%	38.3%	10.7%	100.0%
**SC+CCR+PPM+Q+V+AVT+AVP**	10102	28481	13196	41842	10430	104051
	9.7%	27.4%	12.7%	40.2%	10.0%	100.0%

While pharmaceutical interventions did little to redistribute the age-specific burden of influenza infection, non-pharmaceutical interventions appear to shift the burden towards younger age groups. This may be because measures like school closure and community-contact reduction redistribute more infections to the household, where children tend to be more prone to infection than adults. **Table 7** presents the distribution of transmission events by location, along with a calculation of the percentage of all cases represented by each location. Results suggest that pharmaceutical measures have little impact on the role of different locations in influenza transmission, but that non-pharmaceutical measures—school closures in particular—redistribute infection events to the household and community.

**Table 7:** Transmission events by location, and percentage of total infections accounted for by each location.

Intervention	Location			
	Household	School/work	Community	Total
**None**	337,135	233,370	107,040	677,546
	49.8%	34.4%	15.8%	100.0%
**V+AVT**	309,759	214,515	98,407	622,681
	49.7%	34.5%	15.8%	100.0%
**V+AVT+AVP**	298,522	206,797	95,075	600,394
	49.7%	34.4%	15.8%	100.0%
**CCR+PPM+VI**	101,447	71,084	31,240	203,771
	49.8%	34.9%	15.3%	100.0%
**CCR+PPM+VI+AVT**	99,827	69,962	30,748	200,537
	49.8%	34.9%	15.3%	100.0%
**SC+CCR+PPM+Q**	105,416	42,435	41,165	189,016
	55.8%	22.5%	21.8%	100.0%
**SC+CCR+PPM+Q+V+AVT+AVP**	57,076	24,453	22,522	104,051
	54.9%	23.5%	21.6%	100.0%

**Fig 2:**
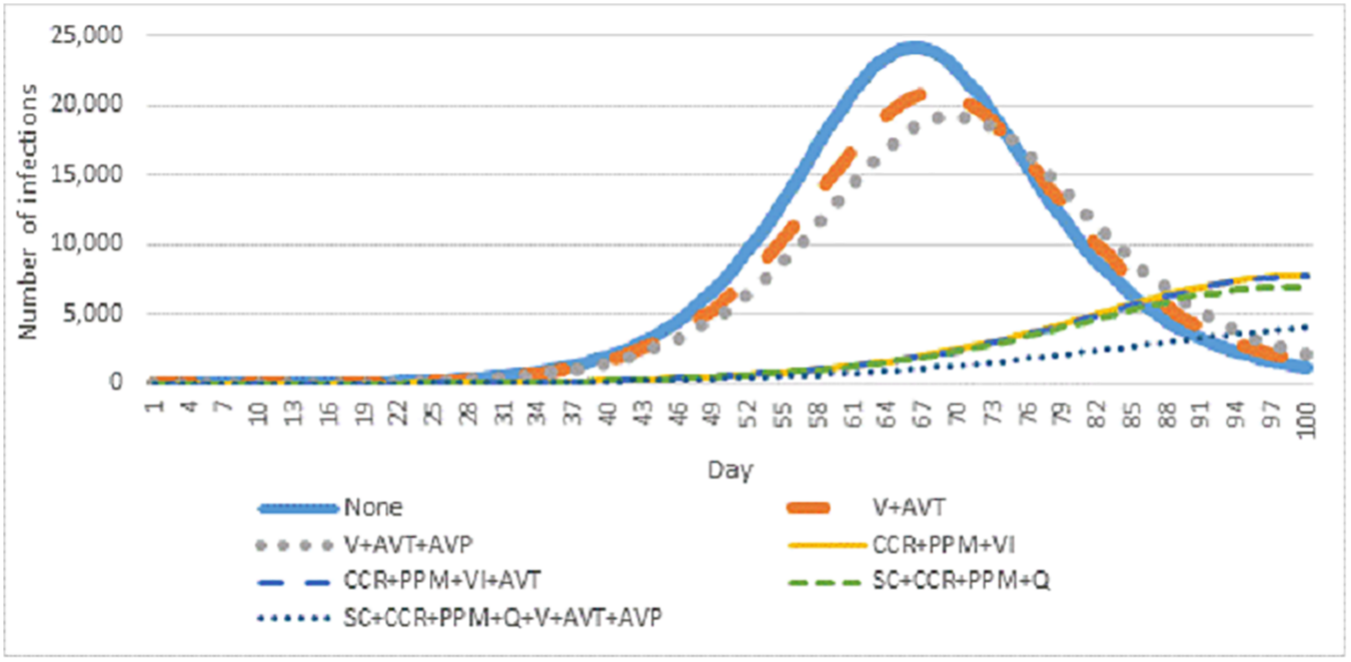
New infections by day under seven key intervention scenarios.

**Fig 2** presents the number of new infections by day over the first 100 days of a pandemic influenza outbreak. Of interest is that pharmaceutical interventions alone (orange and grey lines) appear to result in a contraction of the pandemic peak without affecting its shape; under cases of pharmaceutical intervention—as with no intervention—transmission begins to accelerate about a month after infected individuals were added to the simulation, and there is only a small delay in the pandemic peak. Conversely, aggressive non-pharmaceutical interventions, which implement multiple containment measures in parallel, can delay the pandemic beyond the assumed window for the pandemic wave. Personal protective measures and voluntary isolation seem to account for the majority of this effect, with only small changes resulting from the further addition of community-contact reduction, school closure, quarantine or pharmaceutical measures.

### 3.2 Hospitalization

A total of 2,472 pandemic-associated hospitalizations were projected under a “no intervention” scenario. As seen in **Table 5**, both pharmaceutical and non-pharmaceutical interventions were effective in reducing the number and rate of hospitalizations. As a result, there was substantial variation in the number of hospitalizations that could arise under different intervention scenarios, ranging from 2,472 (no intervention) to 108 (all eight interventions). Hospitalization projections for all 192 intervention scenarios are presented in **S3 Table**.

**Table 8** presents the age-stratified hospitalization totals across the seven intervention bundles of interest. Infants and seniors experienced a disproportionate number of hospitalizations given their population sizes, accounting for 7.4% and 28.8% of hospitalizations despite representing 5.7% and 12.6% of the total population, respectively. Children and adults had disproportionately low hospitalization rates, accounting for 4.2% and 7.8% of hospitalizations, despite representing 16.4% and 15.3% of the population, respectively. While pharmaceutical interventions did little to redistribute the hospitalization burden—as they were not modelled to target particular age groups—non-pharmaceutical measures resulted in higher relative burdens among infants, children and adults, though total hospitalizations decreased across all age groups.

**Table 8:** Predicted number of hospitalizations, and percentage of total hospitalizations accounted for by each group.

Intervention	Age group					
	Infant	Child	Young adult	Adult	Senior	Total
**None**	184	104	193	1279	712	2472
	7.4%	4.2%	7.8%	51.7%	28.8%	100.0%
**V+AVT**	61	34	64	423	234	816
	7.5%	4.2%	7.8%	51.8%	28.7%	100.0%
**V+AVT+AVP**	58	32	60	398	218	766
	7.6%	4.2%	7.8%	52.0%	28.5%	100.0%
**CCR+PPM+VI**	71	30	40	339	154	634
	11.2%	4.7%	6.3%	53.5%	24.3%	100.0%
**CCR+PPM+VI+AVT**	63	26	35	300	136	560
	11.3%	4.7%	6.2%	53.6%	24.3%	100.0%
**SC+CCR+PPM+Q**	74	45	40	234	158	550
	13.4%	8.2%	7.2%	42.5%	28.7%	100.0%
**SC+CCR+PPM+Q+V+AVT+AVP**	15	8	7	48	30	108
	13.4%	7.5%	6.9%	44.4%	27.8%	100.0%

**Fig 3:**
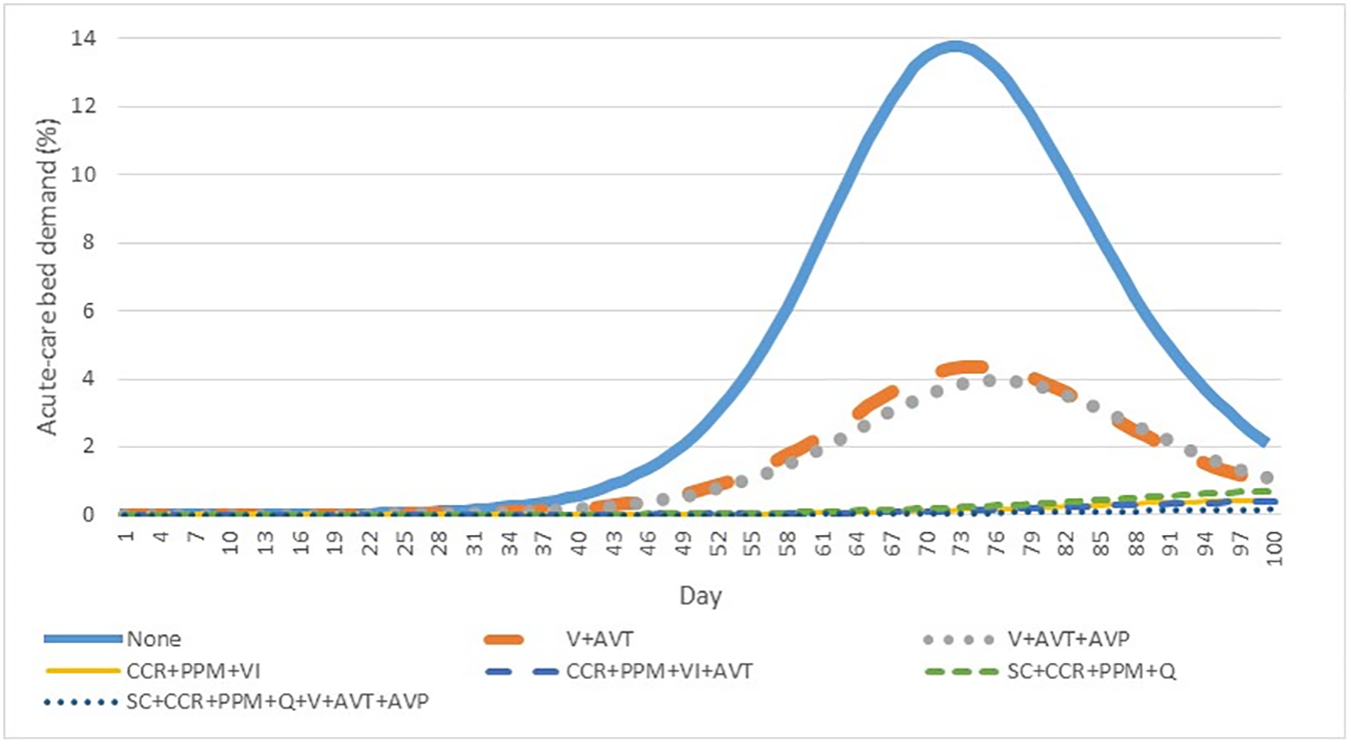
Daily acute-care hospital demand, charted as the percentage of all acute-care hospital beds in the Ottawa–Gatineau CMA, under seven key intervention scenarios.

**Fig 3** presents findings relating to the demand for acute-care hospital beds over the first 100 days of a pandemic influenza outbreak. Similar to the observed effects on symptomatic infections, pharmaceutical interventions resulted in a contraction of peak demand, while layered non-pharmaceutical interventions resulted in its delay and attenuation. Peak acute care demand ranged from 13.8% (no intervention) to 0.2% (all eight interventions). The peak hospitalization demand for all 192 intervention scenarios is summarized in **S4 Table**.

### 3.3 Intensive care unit admission

In a pandemic where no intervention measures are implemented, InFluNet simulations projected approximately 580 ICU admissions over the first 100 days of the outbreak. As with acute-care hospitalization, there is a broad range of ICU demand scenarios, dependent on the intervention scenario being simulated; total ICU admissions ranged from 580 to 26 (all eight interventions). Many moderate intervention scenarios combining a pharmaceutical and non-pharmaceutical intervention resulted in a range of 100 to 400 ICU admissions. The ICU admission estimates for all 192 intervention scenarios are presented in **S5 Table**.

**Table 9** presents estimates of age-stratified ICU admission, alongside a calculation of the proportion of total admissions accounted for by each age group. As with acute-care hospitalization, infants and seniors represented a disproportionately high share of the ICU admissions, reflective of our assumption that infants and seniors were more likely to experience critical illness as a result of complicated influenza infection. This distinction was even more pronounced in infants, which represented 11.9% of ICU admissions under a “no intervention” scenario, despite representing only 5.7% of the total population. Similar to acute-care hospitalization, pharmaceutical measures were not found to significantly affect the distribution of age-specific ICU admission; non-pharmaceutical measures redistributed admissions towards younger age groups, while reducing the total number of admissions.

**Table 9:** Predicted number of ICU admissions, and percentage of total admissions accounted for by each group.

Intervention	Age group					
	Infant	Child	Young adult	Adult	Senior	Total
**None**	69	37	47	309	119	581
	11.9%	6.4%	8.1%	53.2%	20.5%	100.0%
**V+AVT**	23	12	15	102	39	191
	12.0%	6.3%	7.9%	53.4%	20.4%	100.0%
**V+AVT+AVP**	21	11	15	96	37	180
	11.7%	6.1%	8.3%	53.3%	20.6%	100.0%
**CCR+PPM+VI**	24	10	9	81	27	151
	15.9%	6.6%	6.0%	53.6%	17.9%	100.0%
**CCR+PPM+VI+AVT**	21	8	8	71	24	132
	16.0%	5.7%	6.1%	54.0%	18.3%	100.0%
**SC+CCR+PPM+Q**	33	18	9	52	20	132
	25.0%	13.6%	6.8%	39.4%	15.2%	100.0%
**SC+CCR+PPM+Q+V+AVT+AVP**	7	3	2	11	4	27
	25.9%	11.1%	7.4%	40.7%	14.8%	100.0%

**Fig 4:**
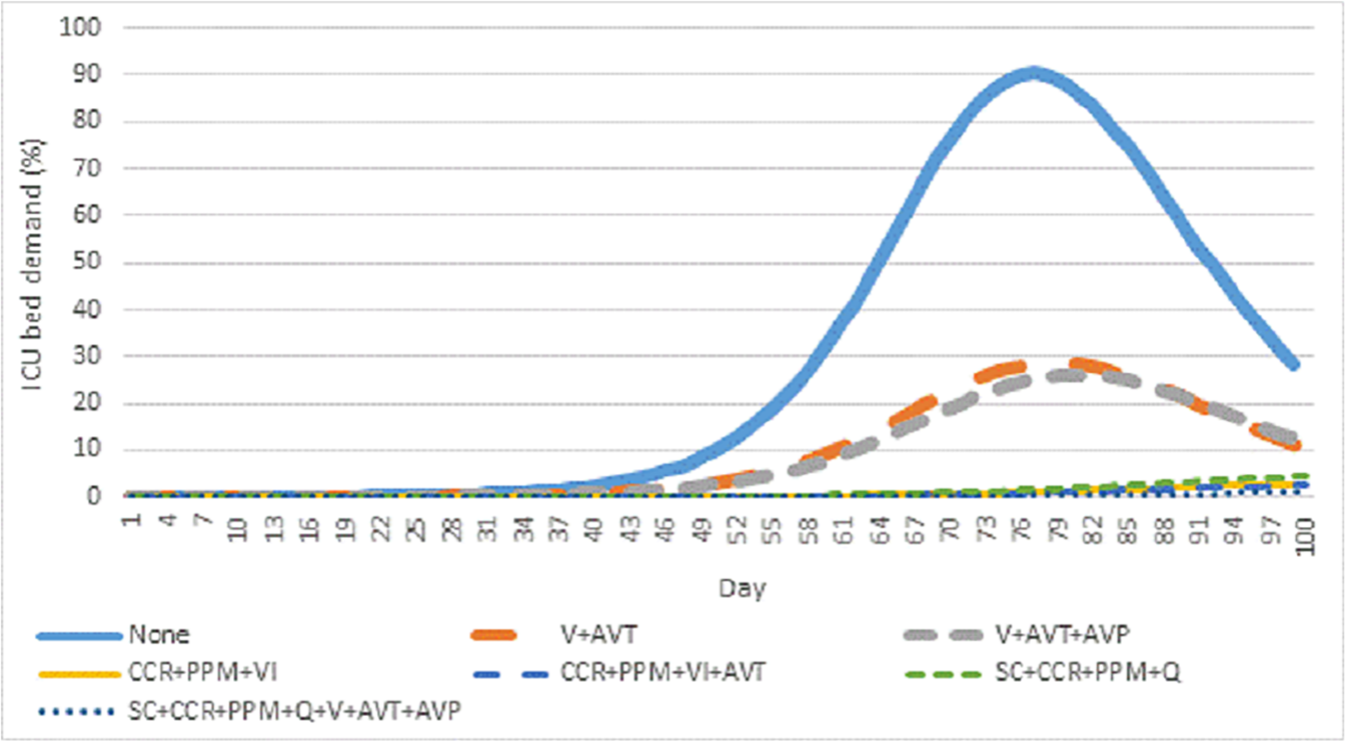
Daily ICU demand, charted as the percentage of all ICU beds in the Ottawa–Gatineau CMA, across seven interventions of interest.

**Fig 4** presents the findings relating to daily demand for ICU beds, as a percentage of the Ottawa–Gatineau CMA ICU capacity. Again, pharmaceutical measures resulted in a contraction of the pandemic peak, while layered non-pharmaceutical measures produced a dramatic attenuation of the wave itself. Peak ICU demand ranged from 90.2% (no interventions) to 1.1% (all eight interventions). The peak ICU demand for all 192 intervention scenarios is summarized in **S6 Table**.

### 3.4 Mortality

The InFluNet model projected 363 pandemic-related deaths under a “no intervention” scenario. Rigorous non-pharmaceutical interventions and most intervention scenarios incorporating vaccination reduced this figure to below 100, while scenarios modelling antiviral treatment, prophylaxis and more moderate non-pharmaceutical interventions tended to predict between 100 and 300 deaths. The mortality estimates for all 192 intervention scenarios are presented in **S7 Table**.

Age-specific mortality rates are presented in **Table 10**, alongside the proportion of the total mortality estimate for each age group. Seniors are over-represented with regard to mortality, accounting for 41.2% of deaths in the “no intervention” scenario. This was due in part to the assumption that additional mortality would occur in seniors outside of the hospital setting and reflects the assumption that many influenza deaths will occur among those with weaker immune systems. Interventions had relatively little effect on the age-specific distribution of mortality, suggesting that age-specific mortality rates dominate mortality distribution, rather than rates of symptomatic infection. Because influenza-related mortality is such a rare occurrence, even significant shifts in age-specific infection rates had relatively little effect on mortality distributions.

**Table 10:** Predicted number of deaths, and percentage of total mortality accounted for by each age group.

Intervention	Age group					
	Infant	Child	Young adult	Adult	Senior	Total
**None**	4	4	27	179	150	364
	1.1%	1.1%	7.4%	49.2%	41.2%	100.0%
**V+AVT**	1	1	9	58	49	118
	0.8%	0.8%	7.6%	49.2%	41.5%	100.0%
**V+AVT+AVP**	1	1	8	54	45	109
	0.9%	0.9%	7.3%	49.5%	41.3%	100.0%
**CCR+PPM+VI**	1	1	4	35	25	66
	1.5%	1.5%	6.1%	53.0%	37.9%	100.0%
**CCR+PPM+VI+AVT**	1	1	4	31	22	59
	1.7%	2.4%	6.7%	52.2%	37.0%	100.0%
**SC+CCR+PPM+Q**	1	1	4	24	25	55
	1.8%	1.8%	7.3%	43.6%	45.5%	100.0%
**SC+CCR+PPM+Q+V+AVT+AVP**	0	0	1	5	5	11
	0.0%	0.0%	9.1%	45.5%	45.5%	100.0%

**Fig 5:**
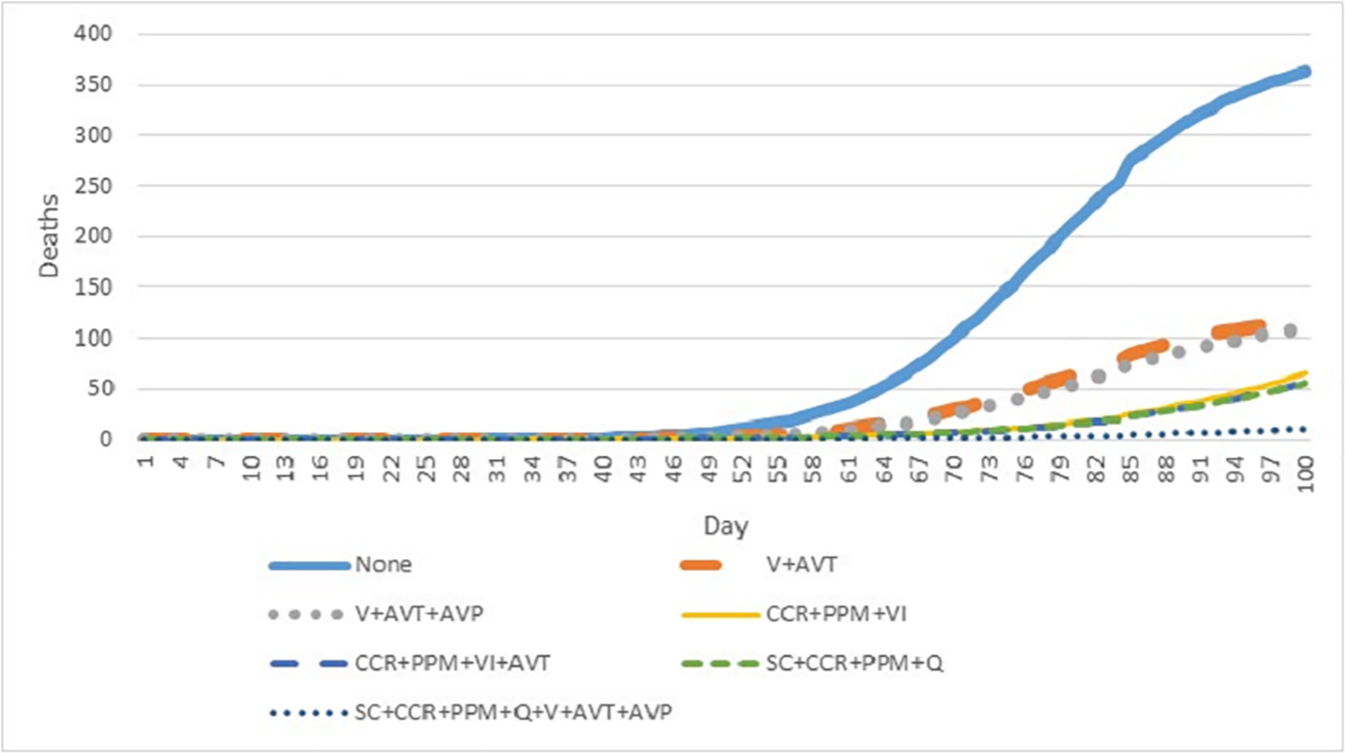
Cumulative mortality over the first 100 days of a pandemic influenza outbreak across seven interventions of interest.

**Fig 5** presents the cumulative number of deaths projected to occur in the first 100 days of a pandemic outbreak across the seven interventions of interest. Deaths begin accumulating almost two months after initial seeding of infected individuals. While mortality totals in “no intervention” and pharmaceutical intervention scenarios appeared to be levelling off after 100 days, non-pharmaceutical interventions did not demonstrate similar rate reductions, suggesting a threat of a prolonged first wave or more severe second wave.

### 3.5 Economic analysis

Estimates of the economic burden for an influenza pandemic—for the seven intervention scenarios of interest—ranged from CAD $115 million to $2.15 billion in the Ottawa-Gatineau CMA alone. The cost breakdown for each intervention scenario is presented in **Table 11**, with more detailed, intervention-specific summaries available in **S8 Table**. Of the interventions that were subject to economic evaluation, a layered non-pharmaceutical approach, in combination with antiviral therapy, seemed to reduce the overall economic burden the most. Intervention costs per life-year saved are presented in **Table 12.** Vaccination appears to be the most cost-effective approach, followed by other pharmaceutical measures, voluntary isolation and personal protective measures. Interestingly, there does not appear to be substantial cost-effectiveness either in combining antiviral therapy and prophylaxis or adding antiviral therapy to a layered non-pharmaceutical intervention. The least cost-effective approaches incorporated school closures, which resulted in massive costs associated with lost school and work days, with relatively little additional benefit in terms of life-years saved.

**Table 11:** Predicted costs (CAD) of pandemic-associated morbidity and mortality, as well as key intervention inputs.

Cost category	Intervention						
	None	V+AVT	V+AVT+AVP	CCR+PPM+VI	CCR+PPM+ VI+AVT	SC+CCR+ PPM+Q	SC+CCR+PPM+Q+V+AVT+ AVP
Hospital bed days	10,303,296	3,396,920	3,188,520	2,642,512	2,334,080	2,292,400	450,144
ICU bed days	12,087,200	4,001,280	3,751,200	3,146,840	2,771,720	2,750,880	541,840
Deaths (infant)	9,420,688	2,355,172	2,355,172	2,355,172	2,355,172	2,355,172	0
Deaths (child)	8,830,976	2,207,744	2,207,744	2,207,744	2,207,744	2,207,744	0
Deaths (young adult)	52,830,738	17,610,246	15,653,552	7,826,776	7,826,776	7,826,776	1,956,694
Deaths (adult)	245,961,394	79,696,988	74,200,644	48,093,010	42,596,666	32,978,064	6,870,430
Deaths (seniors)	63,644,400	20,790,504	19,093,320	10,607,400	9,334,512	10,607,400	2,121,480
Lost school days	72,194	23,514	21,860	5,044,494	4,962,104	910,837,138	794,463,188
Lost work days (YA + adults)	1,819,212	600,371	566,482	38,086,197	37,429,987	1,179,410,918	1,023,034,132
Lost work days (seniors)	83,759	25,609	23,876	583,812	572,259	599,601	314,242
Vaccinations	0	8,724,240	8,724,240	0	0	0	8,724,240
Antivirals	0	7,783,530	9,048,375	0	2,506,608	0	2,593,125
Masks	0	0	0	747,792	747,792	747,792	747,792
Total	405,053,858	147,216,118	138,834,986	121,341,749	115,645,420	2,152,613,885	1,841,817,306

**Table 12:** Predicted life-years lost and cost per life-year saved, by intervention

Intervention	Life-years lost	Cost per life-year saved (relative to no intervention)
No intervention	9,421	N/A
V+AVT	3,026	$2,581
V+AVT+AVP	2,801	$2,685
CCR+PPM+VI	1,767	$6,671
CCR+PPM+VI+AVT	1,607	$6,752
SC+CCR+PPM+Q	1,393	$260,472
All interventions	267	$199,888

### 3.6 Sensitivity analysis

The sensitivity analyses detailed in **S9 Table** produced three main findings. First, personal protective measures, voluntary isolation, quarantine and vaccination all demonstrated a wide range of potential outcomes, depending on parameter assumptions. School closures, community-contact reduction, antiviral therapy and antiviral prophylaxis exhibited relatively little change between BG, WC and BC scenarios. Vaccination and personal protective measures showed particularly high sensitivity to shifting assumptions, with all six health outcome counts shifting over 100% between BC and WC scenarios.

Second, interventions to interrupt community transmission became less effective as transmissibility increased. As shown in **Table 13**, all interventions produced a smaller reduction in the number of symptomatic cases relative to no intervention under a higher transmissibility parameter, though the order of interventions in terms of effectiveness did not change. The differential impact between BG, BC and WC intervention scenarios was also smaller at higher disease transmissibility, though a similar effect was not observed at a higher hospitalization rate.

**Table 13:** Percent (%) reduction in number of symptomatic cases, given influenza transmissibility parameter equivalent to R_o_ of 1.65 or 1.80.

Intervention	Transmissibility parameter (τ)	
	τ = 0.275	τ = 0.3
**SC**	-1.1	-0.8
**CCR**	-0.6	-0.4
**PPM**	-16.4	-8.8
**VI**	-36.5	-27.5
**Q**	-37.7	-28.2
**V**	-7.9	-6.1
**AVT**	-0.2	-0.1
**AVP**	-2.8	-0.9

Third, increasing the hospitalization rate had a much more dramatic effect on health outcomes than did increasing disease transmissibility. **Table 14** presents the “no intervention” findings for the four scenarios. The higher transmissibility in Scenario 2 led to more infections, but only relatively small increases in mortality and hospital-resource use. The higher hospitalization rate in Scenario 3, however, led to large increases in predicted hospital resource demand and mortality relative to Scenario 1, despite having the same transmissibility and fewer symptomatic cases.

**Table 14:** Health outcome summaries for four pandemic scenarios [Scenario 1: τ = 0.275; hospitalization rate = 0.4%; Scenario 2: τ = 0.3; hospitalization rate = 0.4%; Scenario 3: τ = 0.275; hospitalization rate = 1.0%; Scenario 4: τ = 0.3; hospitalization rate = 1.0%].

Outcome	Scenario			
	1	2	3	4
Symptomatic cases	677,546	713,920	675,699	712,553
Hospitalizations	2,472	2,633	4,893	5,217
ICU	580	612	1,149	1,200
Peak hospital demand (%)	14	16	27	32
Peak ICU demand (%)	90	103	179	204
Deaths	363	400	717	791

## 4. Discussion

The objective of this study was to assess the threat posed by the emergence of a novel, transmissible pandemic influenza strain, with respect to potential health and economic burdens. A key area of focus was the assessment of the adequacy of hospital-resource capacity to accommodate expected increases in patient demand, both in acute and intensive-care settings. To accomplish this, we developed and validated InFluNet, a novel mathematical model, which incorporates stochastic elements to account for uncertainty in disease transmission dynamics.

Using a simulated population representative of the Ottawa–Gatineau CMA, we suggest that the timely implementation of a layered, multi-pronged intervention strategy will effectively control pandemic transmission and protect hospital resource adequacy. However, even aggressive intervention simulations produced illness attack rates over 10% and over 100 deaths. The economic burden is expected to be high, estimated between CAD $115 and $405 million, with costs rising into the billions when extended school closure is implemented. Our model suggests that the most cost-effective approach to pandemic control is early pandemic vaccination combined with antiviral therapy and prophylaxis. However, a review of the results from all 192 scenarios (**S2–S7 Tables**) showed a steep diminishing return on investment associated with the addition of antiviral treatment and prophylaxis to an effective vaccination campaign, suggesting that much of the cost-effectiveness of pharmaceutical interventions is driven by strong vaccination campaigns. In situations where vaccines may be unavailable or in short supply, a combination of community-contact reduction, personal protective measures, voluntary isolation and antiviral therapy was also found to be highly cost-effective. However, our estimates did not account for the potential costs of community-contact reduction, which may include shifts in consumer behaviour.

Vaccination, personal protective measures and isolation of infected individuals were found to be the most effective interventions, whereas school closure, community-contact reduction, antiviral therapy and antiviral prophylaxis had less effect on pandemic burden. Sensitivity analysis suggested that the most effective interventions were also those most susceptible to change under shifting assumptions of effectiveness, adherence and timing. All interventions became less effective in limiting transmission as disease transmissibility increased. In all cases, delayed interventions were significantly less effective than cases in which the intervention was implemented from the start of the simulation, suggesting the need for strong and proactive preparedness planning. Sensitivity analysis also suggested that a more virulent strain, with a higher hospitalization rate, is of greater concern than a more transmissible one, with a hospitalization rate of 1% threatening to overwhelm hospital resource capacity even under moderate transmissibility assumptions.

Pharmaceutical and non-pharmaceutical interventions resulted in different effects on the evolution and progression of the pandemic. While pharmaceutical interventions did little to alter the location of transmission events or age-specific burden, non-pharmaceutical interventions tended to redistribute more transmission events to the household and community, as well as to younger age groups. This may be because we did not model age-specific targeting of pharmaceutical interventions and assumed that young adults and adults had a higher community-contact rate than did other age groups; as a result, community-contact reduction and voluntary isolation seemed to disproportionately benefit these age groups.

A common simplification in modelling studies is the assumption of homogenous mixing, wherein individuals are assumed to interact equally across age and geographic groups [96]. This is problematic, as it can overestimate the final pandemic size, leading to unrealistic predictions of hospital-resource strain and the scale of intervention required for transmission containment [41, 97]. Further, it precludes analysis of particular interventions targeted towards specific age groups, such as vaccination campaigns prioritizing children or the elderly. To avoid this, InFluNet models a two-layered system of heterogeneous mixing, wherein individuals in different age groups interact at different rates depending on their location, reflecting the age- and location-dependent forces that will influence disease transmission rates.

Our vaccination simulations assumed that a well-matched pandemic vaccine would be available at the onset of the pandemic; this may represent an unlikely scenario, as vaccine production, development and distribution can take over six months [16, 31]. However, we included this intervention as a means of evaluating the impact of the intervention were it to be available. It should also be noted that logistical challenges, vaccine hesitancy and limited stockpiles of consumables can further constrain the achievement of optimal coverage levels. The effectiveness of timely vaccination suggests value in strengthening international collaboration with regard to surveillance and sharing of circulating influenza strains. Also, by assuming that older age groups maintain some immunity to the pandemic strain, we are modelling the emergence of a pandemic strain that is not entirely novel: this reflects the experience of the past four pandemics but could underestimate population susceptibility to an entirely novel strain, such as an avian influenza. It should also be noted that antiviral prophylaxis may only be available in the early stages of an outbreak, after which contract tracing may become infeasible [30]; we do not account for this in our model.

Pharmaceutical interventions tended to reduce the overall burden of the pandemic without affecting its timing. Non-pharmaceutical interventions, by contrast, tended to delay the development of the pandemic to an extent that the pandemic was not completed after 100 days. We used 100 days as the upper limit of pandemic-wave duration, but such containment of transmission could lead to either a prolonged wave or more severe second wave. This is because pharmaceutical measures contribute to shifting the susceptibility profile of a population, whereas non-pharmaceutical measures contain transmission without affecting the population profile. As a result, if non-pharmaceutical measures are retracted prematurely, there is the risk of disease re-emergence.

The present study is subject to certain limitations. First, we do not account for possible adverse side effects from pharmaceutical interventions, which may marginally reduce the health benefit of mass vaccination [33] and antiviral prophylaxis [98]. We assumed that these associated risks would be insignificant in the context of other unavoidable uncertainties in model assumptions.

Second, we treat the entire CMA as a single homogenous area. While individuals are likely to mix preferentially in neighbourhood and workplace pockets, we decided that the most effective method of evaluating macro-level threats to the community and health system would be to treat the CMA as a single unit.

Third, we have excluded analysis of preferential targeting of at-risk individuals—and associated ethical considerations—as outside the scope of this analysis, prioritizing instead an assessment of community-level practices to control pandemic burden.

Fourth, we do not include every conceivable intervention, excluding for example hospital triage protocols and influenza helplines. Hospital triage and influenza helplines were excluded because we view their main benefit as being a reduction in unnecessary hospital visitation, and our hospitalization rate already assumed that only those needing hospitalization would be admitted. However, we note that triage protocols could increase the proportion of individuals—especially seniors—that die outside of hospital settings. We also did not model nosocomial infection and therefore did not examine the value of hospital-infection control practices.

Fifth, we chose to model social-contact behaviour based upon empirical data from the United States, as none were available in Canadian contexts. While this may skew our estimates slightly, similar contact-rate assumptions in previous modelling studies of the mid-sized Ontario cities London [39] and Hamilton [33] suggest that the same contact structure would apply in Ottawa.

Sixth, while agent-models (ABMs) are more effective at modelling the stochasticity inherent in disease transmission, the decision was made to pursue a differential equation models (DEMs). ABM models require a large computational burden and, given the number of InFluNet compartments alongside the age-specific and location-specific transmission dynamics, an ABM would have obstructed the depth of analysis, limited sensitivity analysis and impeded its uptake among public-health practitioners with limited modelling training [99]. As a secondary objective of this project was to construct a model that was scalable to different populations and accessible to policy audiences, and considering the output differences between DEMs and ABMs are small within the larger scope of parameter uncertainty, we chose to construct a DEM.

Lastly, as with all prospective mathematical models, InFluNet is subject to a high degree of uncertainty; this is particularly true in our case, where a high level of analytical complexity necessitates numerous assumptions relating to disease and intervention characteristics. We therefore emphasize that the results of our model should be interpreted with caution and are best interpreted as general patterns of intervention effectiveness rather than specific predictions of the number of likely cases, hospitalizations and deaths. Future research can add to this analysis through in-depth assessment of targeted interventions, based on age or risk profile and evaluation of other communities to assess community characteristics that may lead to higher pandemic burdens and strain on health system capacity.

The strength of InFluNet is its incorporation of empirical social contact, disease and intervention data to chart pandemic progression against real-world hospital-capacity data. It also allows the most complex analysis of mitigation strategies, helping to inform pandemic preparedness planning in both community and hospital settings. We are aware of no other mathematical models that incorporated such diverse intervention bundles alongside our broad range of health and economic endpoints. The results of this initial analysis suggest that the hospital capacity of the Ottawa–Gatineau region will be adequate to accommodate a transmissible but mild pandemic influenza under most intervention strategies but that it could quickly become overwhelmed by a more virulent strain.

## 5. Conclusion

This study provides valuable new insights in pandemic preparedness, presenting a novel model of pandemic transmission as it relates to health and economic outcomes and hospital-surge capacity. Our analysis suggests that personal protective measures, isolation of infected individuals and vaccination are most effective at containing pandemic transmission. Even in situations where vaccines are unavailable, a layered approach incorporating the timely implementation of multiple non-pharmaceutical interventions is likely to effectively contain pandemic transmission and maintain the adequacy of hospital resource capacity in the Ottawa–Gatineau area. However, we found that even small increases in disease transmissibility or virulence constitute a significant threat, both in terms of surges in patient demand and overall burden. Given the need for timely interventions, future studies are needed to assess optimal intervention strategies under a broad range of disease, intervention, population and resource assumptions.

## Supporting information

S1 File InFluNet model description. (PDF)

S1 Table Ottawa–Gatineau CMA profile. (PDF)

S2 Table Results of Basic Analysis: Symptomatic infection. (PDF)

S3 Table Results of Basic Analysis: Acute care hospital admissions. (PDF)

S4 Table Results of Basic Analysis: Peak acute care hospitalization demand. (PDF)

S5 Table Results of Basic Analysis: Intensive care unit admissions. (PDF)

S6 Table Results of Basic Analysis: Peak ICU demand. (PDF)

S7 Table Results of Basic Analysis: Mortality. (PDF)

S8 Table Economic analyses for seven intervention bundles of interest. (PDF)

S9 Table Results of sensitivity analysis. (PDF)
