# Supplementary material for: Modelling community-control strategies to protect hospital resources during an influenza pandemic in Ottawa, Canada
Source: PLoS One. 2017 Jun 14;12(6):e0179315. doi: 10.1371/journal.pone.0179315 (PMC5470707; doi:10.1371/journal.pone.0179315)
Supplement: S1 File — (PDF) [file pone.0179315.s001.pdf]

S1 File: InFluNet model description

S1.1 Social contact network

Although it is common for models to combine location-specific estimates of transmission dynamics [1-3], this risks obscuring the setting within which the outbreak is being propelled forward, as well as analyses of how specific interventions will influence outbreak development and severity. InFluNet models a transmission cycle composed of the average time spent in the household (12 hours), at school or work (8 hours) and in the community (4 hours). These baseline time-location divisions are derived from an empirical study of a representative North American municipality [4] and Statistics Canada [5] national estimates of average time spent per day in various locations. They are subject to change under certain interventions, including voluntary isolation, quarantine and school closure.

The simulation model was developed to predict the behaviour of a pandemic outbreak in a developed, urban setting. Demographic parameters — such as age-stratified population, average earnings and local unemployment rates — mirror those of the Ottawa–Gatineau census metropolitan area (CMA) from the 2011 census [6]. The household contact group size was assumed to be equal across all households, and was calculated as the mean size reflected in the Census data. School and work group sizes were estimated to constitute thirty and twenty people, respectively, choices that were informed by similar modelling approaches [7, 8]. The community group size was fixed at 100 people, reflecting the number of random contacts an individual is likely to have in their home and work neighbourhoods [9, 10].

Because the expected duration of a pandemic outbreak is short, we assumed a closed population, with no birth or death rate included, as these are unlikely to meaningfully affect results [15]. While past modelling studies have incorporated assumptions of heterogeneous mixing, few base these assumptions on empirical data, as is the case with InFluNet [15].

The age-specific numbers of daily contacts in **Table S.1** have been estimated from past empirical studies in the United States [4, 15]. These studies reported the total number of contacts within and between age groups. We calculate per-capita contact rates by dividing the total contacts by the total population of each age group, calibrating estimates to reflect the empirical data of overall contacts to within 0.5 contacts per day. Between-group contact rates are asymmetrical as a result of differences in the population size of each age-group. For example, every infant is likely to interact with an adult on a daily basis, but not every adult is likely to have daily interaction with an infant.

Table S1. Average number of daily contacts by age group per person per day [4].

	Infant	Child	Young adult	Adult	Senior	Total
**Infant**	0.9511	3.5509	1.6740	4.8698	0.6594	11.7052
**Child**	1.2237	7.3670	1.6153	3.5244	0.6363	14.3668
**Young adult**	0.6096	1.7070	6.7059	12.1926	1.3209	22.5359
**Adult**	0.6195	1.3010	4.2591	12.6380	1.4094	20.2271
**Senior**	0.3498	0.9794	1.9239	5.8766	2.1827	11.3124

These daily totals are further divided into location-specific and age-stratified contact tables, through estimation of how the location-specific frequency and intimacy of interaction will vary between age groups (**Tables S.2–S.4**). These estimates are used to generate hourly contact rates,

which determine the number of effective contacts that will in turn influence risk of disease exposure and contraction [16]. Contact rates are subject to change under various interventions, including community-contact reduction and personal protective measures. It should be noted that we assumed all households were of equal sizes, as informed by a previous influenza model [17]; this prevented an assessment of clustering, which would have been possible in an agent-based model. As clustering is a rarer phenomenon in highly transmissible pandemic influenza scenarios, relative to diseases with dominant high-risk groups such as sexually transmitted diseases, we did not feel that this assumption will significantly affect our final outputs [18]. We recognize that the assumption of random contacts between groups may have implications for early-stage predictions relative to a model with a more heterogeneous household mixing structure. However, as our focus is on peak resource demand and final epidemic size, differences in initial transmission patterns were deemed acceptable.

Table S2. Number of contacts by age group per day (household)

	Infant	Child	Young adult	Adult	Senior	Total
**Infant**	0.6658	2.4856	1.1718	3.4088	0.5276	8.2596
**Child**	0.8566	1.8417	1.1307	2.4671	0.5091	6.8053
**Young adult**	0.4267	1.1949	1.3412	1.8289	0.5283	5.3200
**Adult**	0.4337	0.9107	0.6389	3.7914	0.7047	6.4793
**Senior**	0.2798	0.7835	0.7695	2.9383	1.3096	6.0808

Table S3. Number of contacts by age group per day (school and workplace)

	Infant	Child	Young adult	Adult	Senior	Total
**Infant**	0.1427	0.5326	0.2511	0.7305	0.0659	1.7228

**Child**	0.1836	4.4202	0.2423	0.5287	0.0636	5.4383
**Young adult**	0.0914	0.2560	3.3530	7.9252	0.3302	11.9558
**Adult**	0.0929	0.1951	2.7684	5.0552	0.3523	8.4641
**Senior**	0.0350	0.0979	0.4810	1.4691	0.3274	2.4104

Table S4. Number of contacts by age group per day (community)

	**Infant**	**Child**	**Young adult**	**Adult**	**Senior**	**Total**
**Infant**	0.1427	0.5326	0.2511	0.7305	0.0659	1.7228
**Child**	0.1836	1.1050	0.2423	0.5287	0.0636	2.1232
**Young adult**	0.0914	0.2560	2.0118	2.4385	0.4623	5.2601
**Adult**	0.0929	0.1951	0.8518	3.7914	0.3523	5.2837
**Senior**	0.0350	0.0979	0.6734	1.4691	0.5457	2.8211

More sophisticated interactions such as concurrent relationships and social biases in mixing were excluded, as the main focus of the model is to assess the cumulative and peak burden of an influenza pandemic, with particular emphasis on the adequacy of hospital surge capacity during times of peak patient demand; the above assumptions are more important in the early stages of an outbreak and have been rarely incorporated in past disease-transmission models [15]. InFluNet should adequately reflect the heterogeneity of social mixing to estimate trends in disease-transmission patterns.

S1.2 InFluNet Transmissibility

Realistic modelling of the rate of disease transmission is critical to obtaining useful results and insights. A problematic assumption in infectious disease modelling is to chart disease transmission

as driven by the basic reproduction number (R_0) or the effective contact rate (β). R_0 represents the number of secondary infections that an average index case would produce in a completely susceptible population. Typically, if $R_0<1$, an outbreak does not occur, whereas if $R_0>1$, an outbreak will occur. In simple infectious disease models with no background death rate, the equation for the reproduction number is as follows:

$$R_0 = \gamma\beta T, \qquad \text{(S1)}$$

where γ is the number of "effective" contacts per unit time, β is the per-contact probability of infection transmission and T is the average duration of the infectious period.

This method of modelling disease transmission introduces error in several ways [19]. First, as soon as the first infection occurs, the population is not entirely susceptible, so R_0 no longer applies, and a related variable, the effective reproduction number (R_e) becomes relevant. The same is true once interventions are implemented. Second, an estimated R_0 has been found to vary depending on the method of calculation [20], and the same model can give notably different estimates depending on the method of calculation used [21]. One study, for example, used four methods to estimate the R_0 of the 1918 Spanish flu pandemic: estimates ranged from 2.1 to 2.98 (95% CI 0.5–3.5) [22].

The effective contact rate, a combination of the number of contacts and the per-contact transmission probability, is also problematic for model analysis. It is a simplification of transmission dynamics that incorporates numerous factors, and grouping them into a single rate precludes analysis of how these factors, and interventions acting upon them, can affect the timing and scale of an outbreak. Instead, InFluNet uses a "next-generation operator" approach described previously in a model of smallpox [15] and reflective of a heterogeneous population [23].

The number of contacts per unit time (γ) among individuals within and across different age groups is defined by the population contact model, reflecting preferential mixing between certain age groups, and is influenced by mixing group size and location. The probability of transmission given a contact is the result of the susceptibility of the susceptible group (α), infectivity of the infected group (η) and duration of contact between the two (σ). The baseline assumption is that 5% of adults and 35% of seniors will not be susceptible to pandemic infection due to prior exposure, a rate of pre-existing immunity observed in the United States in a study of the 2009 H1N1 pandemic [24]. In the absence of reliable data, we assume that all infected individuals will be fully infectious; both parameters can change according to vaccination and treatment status. An important value of pharmaceutical interventions is that only they can alter the susceptibility and infectivity profile of a population, whereas non-pharmaceutical interventions act instead on the social–contact patterns of a population [29]. The mean number of transmission events per unit time (τ) is a parameter reflecting the intrinsic transmissibility of the disease in question; in the case of InFluNet, this has been assumed to range between 0.17 and 0.42, which would mirror an R_0 value between 1.5 and 2.5, reflecting historical estimates of pandemic influenza [25-28]. This approach incorporates sophisticated techniques to model realistic disease-transmission assumptions and allows assessment of how interventions are likely to affect pandemic transmission and burden. In this study, we modelled a transmissibility parameter of 0.275, equivalent to the transmissibility reported from the 1957 pandemic [30]. This pandemic was chosen as it demonstrated a moderate transmissibility, relative to the more severe 1918 pandemic and milder 1968 and 2009 pandemics.

S1.3 Model Structure

The loop structure was determined by calculating the variance between model simulations, and running continuous simulations until the average value had a standard error below 5%.

Susceptible individuals (S) can be vaccinated, moving either to an immune, “vaccinated” group (V) or remaining in a susceptible, “failed vaccination” (S_V) group; the proportion of individuals moving into these will depend on the estimated vaccine coverage (N_C) and vaccine efficacy (V_E). A failed vaccination group was included to reflect the fact that those who get vaccinated but still become infected may experience reduced infectiousness or disease severity than infected individuals who did not receive vaccination [12, 35]. Susceptible individuals can also receive antiviral prophylaxis (AVP), moving to a less susceptible group that, if still infected, will experience a less severe infection. Infection can occur among “susceptible”, “failed vaccination” and “antiviral prophylaxis” individuals, though the severity of influenza infection, both with respect to infectivity and likelihood of complications, may be reduced among the S_V and AVP groups.

The simulation begins with 50 infected cases being seeded in the population across the five age groups at a rate proportional to the population size of each group. When subsequent infections occur, infected individuals move immediately to the latent, or “exposed” (E) group, where they remain for the duration of the influenza latent period €, which is distributed between one and three days (30% one-day; 50% two-day; 20% three-day latent periods), reflecting the assumptions of past models [8, 36] and empirical Canadian data from the 2009 H1N1 pandemic (pH1N1) [37]. Following completion of the latent period, infected individuals pass into an infectious period until they recover (r) after four to seven days (40% four-day; 30% five-day; 15% six-day; and 15% seven-day infectious period), again reflecting the assumptions of past models [8, 12, 13] and empirical Canadian data from the 2009 H1N1 pandemic [38]. Of those who have been infected, two thirds experience symptomatic, “clinical” infection (I_C), while one third develop asymptomatic infections (I_A) and are half as infectious as symptomatic individuals [9, 10, 39-41]. It should be

noted that, in a study of the infectious period of pH1N1 [38], 8% of the infected study subjects were still shedding replicating virus after eight days, so our estimates may underestimate the infectious period for a small proportion of individuals. However, it was decided that this broad distribution — alongside longer durations of infection for complicated, hospitalized cases — was likely to capture what could reasonably be expected during a future pandemic.

Of those who are symptomatic, a proportion can seek treatment with antivirals, reducing their infectivity and hospitalization rate by a certain percentage and shortening their duration of infection by one day [9, 29, 42, 43]. The movement of populations through the healthcare system is derived from approximations of the empirical data from the North American experience of the 2009 pH1N1. A small proportion (0.4–4.0%) [44-46] will require hospitalization in an acute bed for an average of four days, as reported by Canadian Institute of Health Information [47] during pH1N1. Age-specific hospitalization rates are modelled with children as the baseline group and other age groups scaled to the childhood hospitalization rate according to predefined ratios. Infants are expected to have a five-fold higher hospitalization rate, young adults twice as high, adults four times as high, and seniors ten times as high. While it is impossible to know the age-specific hospital burden prior to pandemic emergence, these ratios reflect the empirical hospitalization data gathered in the United States during the 2009 pandemic [48].

The mortality rate for those in an acute bed is 3.125% [49, 50]. Of those hospitalized, 16% will also require ICU care and mechanical ventilation, approximating empirical data from the Canadian experience of pandemic influenza [46]. It also assumes that all those who are admitted to the ICU have a sufficiently critical illness to also warrant ventilation; in the study of the 2009 pandemic in Canada, 93.2% of those who received ICU care also received ventilation [46]. Of those requiring ICU and ventilator care, there is an associated mortality rate of 50% [50]. As with

age-specific hospitalization rates, we model age-specific mortality rates given hospitalization, approximating the assumptions of past studies modelling Canadian contexts: using infants as the baseline group, mortality in children is twice as likely; mortality in young adults and adults is ten times as likely; and mortality in seniors is twenty times as likely [51]. We assume that 100% of deaths in those under 65 years old occur in hospital, while 75% of deaths among seniors occur in hospital, with the remaining 25% in other settings such as retirement and long-term care facilities [52, 53]. The average duration of hospital stay for those requiring ICU care and ventilator support is estimated to be ten days, approximating empirical observations from Canadian experience of pH1N1 [46]. No interventions are modelled that affect the rate of ICU admission, ventilator support, or death given hospitalization. Rather, we model the impact of community interventions in reducing initial infection and clinical disease severity. Descriptions, ranges, and sample values for model parameters are provided in **Table S6**

Table S6. Model parameters

Symbol	Definition	Sample value	References	Range
N_C	Rate of vaccination	$8.5e^{-4}$ (1/days)	[51]	$8.5e^{-4}$ (1/days)
V_e	Vaccine efficiency	65%	[12, 35, 54-56]	40–90%
φ	Reduction in infectivity due to vaccination	35%	[12, 35]	20–50%
ε	Rate of disease progression	1/1.6 days	[8, 36, 37]	1/3–1/7 (1/days)
θ	Rate of hospitalization	Age-dependent	[44-48]	$1e^{-3}$–$1e^{-1}$ (1/days)
r	Rate of recovery	r = 1/4.8<br>Hosp = 1/3.35<br>ICU = 1/10.25	[8, 12, 13, 38]	1/4–1/7 (1/days)
ζ	Rate of treatment/prophylaxis with antivirals	$1e^{-3}$	Assumed	$1e^{-3}$ (1/days)
ω	Reduction in recovery time	1 day	[37, 41, 57]	0–2 days

χ	Death rate in hospital setting	Hosp = $1e^{-3}$<br>ICU = 0.1	[49, 50]	$1e^{-3}$–$1e^{-1}$ (1/days)
ρ	Progression through hospital	ICU = 0.05	[46]	0.05–0.5

S1.4 Resources

Hospital resources are aggregated and estimated at the level of the CMA. Data regarding hospital bed capacity — categorized as "acute", "ICU" and "other" — were obtained from the most recent data from the Canadian Institute for Health Information, reported on January 28, 2016 [58]. These data report on hospital bed capacity by function for all hospitals in Canada, except those in Quebec. Investigators first calculated the number of acute beds in each CMA, followed by the number of ICU beds. The number of "other" beds — including long-term care, psychiatric, and rehabilitative beds — was also calculated to inform a discussion of the potential for bed repurposing in emergency situations, though this type of service disruption would be better avoided through community intervention. As Quebec does not contribute to CIHI information collection, hospital data for this province was sought separately. Data were publicly available through the Ministry of Health and Social Services website, last updated on October 4, 2016 [59], and hospitals were logged into a database individually. Some records contained no in-patient beds and were instead designed for delivery of other social services. These were excluded from the analysis, as it was decided that their inclusion would overestimate in-patient hospital capacity during a pandemic event. Because Quebec hospitals did not report their number of ICU beds, we calculated the proportion of hospital beds outside of Quebec that were designated for the ICU (6.2%) and extrapolated this to Quebec hospitals.

No provincial or national data were available on ventilator capacity. Instead, we assumed that each available ICU bed would have a ventilator available; this assumption is supported by similar per-capita availability of ventilators [60] and ICU beds [61]. Combining these hospital-resource data with the Census demographic data, we generated individual profiles for each Canadian CMA; the Ottawa–Gatineau profile included in **S1** was used for the following analysis.

S1.5 Outcomes

The model generates results relating to health and economic outcomes. Health outcomes summarize consequences in terms of the number of symptomatic cases, hospitalizations, ICU admissions, ventilator demand and deaths by age, location and as a percentage of existing capacity. These data are reported on a daily basis and summarized over the course of the entire outbreak.

Economic analysis is conducted in two ways. First the overall economic burden is calculated by aggregating the influenza- and intervention-associated costs to provide a cost–benefit analysis of intervention strategies of interest. Economic estimates are generated by scaling health and intervention endpoints (as generated by the model) by a per-case economic cost, as given in **Table 9**. This is done to show the potentially large economic burden associated with influenza pandemics, and the capacity of timely intervention bundles to mitigate this impact. Second, the relative cost-effectiveness of different intervention strategies is assessed by calculating the cost per life-year saved, relative to a "no intervention" scenario. It should be noted that, while healthcare-associated costs are calculated in the estimate of overall economic burden, they are excluded from the cost-effectiveness calculation.

The economic cost associated with mortality was calculated by assigning a value of CAD $50,000 for each life-year lost (LYL), calculated using the difference between the midpoint of

each age group and the national life expectancy. A 1.5% per year discounting rate was used, reflecting current views in health economics [62]. The CAD $50,000 valuation per life-year gained or saved represents the commonly accepted threshold below which health interventions are considered to be cost-effective in Canada [63]. This valuation was chosen as a middle-ground between present earning value and the subjective valuation of life; recent government estimates of the latter have ranged between six and nine million dollars (USD) [64]. Subjective valuations are inconsistent, do not recognize the economic value of protecting health among younger individuals and may overestimate the value of life to the extent that it renders measured policy recommendations infeasible. A sole focus on present earning value, meanwhile, may underestimate the value of life — particularly among seniors — by focusing only on economic productivity and potential.

The number of lost school days is calculated by combining results from school-closure days, symptomatic cases and hospitalizations. If school closure is one of the interventions being modelled, the number of school-closure days is multiplied by the number of school-aged children. This is added to the number of children that are likely to miss school due to voluntary withdrawal or quarantine, represented mathematically as the product of the number of symptomatic cases, adherence to isolation recommendations and an estimated five-day withdrawal period. Lastly, each childhood hospitalization is estimated to result in an additional four lost school days, and each ICU admission an additional ten lost school days [47]. Each lost school day, whether due to school closure or childhood illness, is estimated to cost CAD $91.85, as informed by past North American modelling studies which assume that lost school days would be made up during summer break [8, 65].

The number of lost adult work days is calculated as the sum of young adults, adults and seniors who will miss work due to illness, hospitalization or school closure. As with children, symptomatic young adults and adults that voluntary isolate or quarantine themselves are expected to miss five days of work, while those hospitalized are expected to miss an additional four, and those requiring ICU care an additional ten [38, 47]. Meanwhile, 10% of symptomatic seniors — reflecting the Canadian employment rate of those over 65 [66]— that voluntarily withdraw are expected to miss five days of work due to illness, four days if requiring hospitalization, and ten days if requiring ICU care. If school closure is being modelled, a certain percentage of adults will also be forced to stay home to care for their children. The economic cost of lost work days is calculated as the product of the number of lost work days and the average daily earnings of an employee (in the case of the Ottawa CMA, this is CAD $192.55 [67]).

There are three intervention-specific resource requirements that are also incorporated into economic calculations. The number of vaccinations needed is calculated as the product of the total population and the vaccination coverage, reflecting the assumption that only one vaccine dose will be needed. An empirical systematic review previously reported no significant difference in immunity between individuals who received one or two vaccine doses [68]. Vaccines are assumed to cost CAD $20/dose, reflecting calculations from the 2009 pandemic in Ontario [51]. The total number of antiviral courses required is the sum of individuals who receive antiviral treatment and prophylaxis; the cost of antivirals is estimated to be CAD $25/course, as informed by past Canadian influenza studies [7, 51, 69]. The number of masks required is calculated as the product of the total population and adherence to mask recommendations; we assume that each adhering individual will require five masks and estimate a cost of CAD $4/mask from a survey of local prices.

S1.6 Intervention approaches

Interventions can be categorized as either pharmaceutical or non-pharmaceutical, with every iteration presented in a single matrix. Pharmaceutical interventions can affect individual susceptibility, infectivity and hospitalization, while non-pharmaceutical interventions can change where and how individuals are interacting. This method allows consideration of 192 different intervention combinations and millions of unique strategy profiles, providing sophisticated insights into optimal control strategies. In this study, we provide a "Best Guess" (BG), "Worst Case" (WC), and "Best Case" (BC) for each parameter, to generate a range of estimated intervention impacts. Each intervention is discussed separately below.

S1.6.1 Vaccination

Vaccination interventions (V) vary according to the time to vaccine delivery, population coverage and effectiveness with regard to individual susceptibility, infectivity and disease severity (reduction in hospitalization rate). The impact of vaccination occurs two weeks after receipt of the vaccine. The maximum weekly vaccination rate is set as 7.5% of the population [51]. Total coverage is estimated to be 35% (range 25–45%). The BG value approximates the average Canadian influenza vaccine coverage between 2001 and 2012 (34.2%) [70]; the WC value represents the lower bound of the 95% confidence interval from the lowest vaccine coverage reported between 2001 and 2012 (2010: 26.5%) [70]; the BC value is the influenza vaccine coverage reported in Ontario following the first wave of the 2009 H1N1 pandemic [51]. The short timeframe of the simulations — coupled with the capping of vaccination coverage — prevents the vaccinated group from approaching infinity over time.

In these simulations, we are modelling the protective effect of a strongly matched pandemic vaccine. We are not modelling the potential impact of seasonal influenza vaccination, as two independent systematic reviews found no evidence of a significant effect of seasonal flu vaccination in preventing a pandemic flu outbreak [56, 71]; in fact, there has been some suggestion that seasonal vaccination may actually increase pandemic infection, as it precludes seasonal infection and temporary immunity [72-75]. Instead, we model the impact of pandemic vaccination under situations where it is available in time to be effective in controlling the outbreak. Vaccine efficacy for susceptibility (VE_S) is estimated to be 65% (range 40–90%), reflecting the findings of four independent systematic reviews [54-56, 76]; vaccine efficacy for infectiousness (VE_I) is assumed to be 35% (range 20–50%), reflecting past modelling assumptions in the absence of reliable empirical data [12, 35]; reduction in hospitalization rate is estimated to be 60% (range 25–90%), as reported in a systematic review of seasonal vaccine impact on seasonal influenza hospitalization, which was viewed as the most reliable estimate available [77]. These changes are incorporated by scaling the susceptibility (α), infectivity (η) and hospitalization rate (θ) of those vaccinated by the estimated impact. Taken together, these three parameters allow us to include the assumption that vaccinated individuals will be less susceptible to infection and experience less severe and infectious illness if infected.

S1.6.2 Antiviral treatment

Antiviral treatment (AVT) scenarios vary by the coverage and effectiveness in reducing infectivity and disease severity (with respect to hospitalization). Antiviral treatment also reduces the duration of the infectious period by one day [37, 41, 57]. The rate of antiviral distribution was set to be sufficiently high so as to not impede receipt of antivirals by infected individuals, reflecting the assumption that easy access to antivirals through local pharmacies would prevent demand from

exceeding distribution capacity. It is also possible to model the impact of antivirals on a resistant viral strain, which is assumed to reduce drug effectiveness by 50% [9, 29, 42, 43]. In our simulations, we assume a non-resistant pandemic strain, with a reduction in infectivity (η) of 75% (range 57–82%), as reported in past systematic reviews and modelling studies [9, 29, 42, 43, 78]. The reduction in the hospitalization rate (θ) is 10% (0–40%), as reported in a systematic review [79].

S1.6.3 Antiviral prophylaxis

Antiviral prophylaxis (AVP) refers to the preventive — rather than therapeutic — use of antivirals among those who are at high risk of viral exposure or severe disease, due to their relationship or proximity to an infectious case or health status, respectively. As InFluNet does not model preferential care for individuals at high risk of severe infection, we assume that prophylaxis — if implemented — would proceed in a targeted manner, with the distribution of drugs among household, school and work contacts, but not throughout the broader community.

We assume that individuals who receive prophylaxis will have a reduced susceptibility to infection (α) of 30% (range 10–50%), approximating conclusions from a systematic review [55]. Of those who are infected despite prophylaxis, we assume that they will have a reduced infectivity and hospitalization rate equal to that among those who received antiviral treatment.

S1.6.4 School closure

School closure (SC) is considered as an emergency measure that will only take effect when the proportion of the population that is currently symptomatically infected surpasses a predefined threshold. We use the threshold of 0.2%, as informed by a previous study modelling school closure [49]. When SC takes effect, all children (5–18 years old) are removed from the school setting, and

their contact rate is set to zero for that eight-hour period [80, 81]; we assumed no increase in household- or community-contact rate [82]. A certain percentage of adults (30–64 years old) are also redistributed, as some parents will be forced to stay home with their children. We assume that 20% (range 8–33%) of adults would be forced to stay home as a result of school closure, approximating empirical data from school closures in New York City as a result of the 2009 pandemic [83].

S1.6.5 Community-contact reduction

For community-contact reduction (CCR), the contact rate (γ) in the community setting is scaled by a certain percentage across all age groups, reflecting social distancing in the community but not the household, school or work environment. We assume a reduction of 50% (range 25–75%), reflecting assumptions made in past modelling studies, as empirical data were unavailable [7, 9, 40, 41].

S1.6.6 Personal protective measures

Personal protective measures (PPM) include hand hygiene and facemask use, which are modelled as changes in the duration of contacts between individuals (σ). Respiratory etiquette (covering mouth during cough or sneeze) was not included, as studies suggest that it is ineffective in preventing viral transmission [84-87]. Each of these behaviours has an effectiveness measure informed by past research. Hand hygiene is estimated to be 26% (3–44%) effective [88-92] and mask use 60% (8–82%) effective [93-97]. The actual impact of these interventions will depend on the population adherence, which is set by the user. We assume adherence rates of 38% (20–55%) and 3% (1–5%) for hand hygiene and mask use, respectively [88, 98-102].

S1.6.7 Voluntary isolation

Voluntary isolation (VI) defines a percentage of symptomatically infected individuals who will withdraw from school or work settings while ill. Isolated individuals have no contact in the school/work location, though their household and community contacts are assumed to be unchanged [103, 104]. Adherence to voluntary isolation recommendations is estimated to be 30% (range 10–50%), approximating empirical data from the 2009 pandemic [104].

S1.6.8 Quarantine

We present quarantine (Q) as a voluntary withdrawal to the household for the entire duration of infection and view mandatory or hospital quarantine as infeasible in mass-infection scenarios such as pandemic influenza. Quarantined individuals have no contact in the school/work or community setting, though their household contact rate is assumed to be unchanged. In this study, when quarantine is being simulated, it is as a proportion of voluntarily isolated individuals that adhere to more stringent isolation recommendations. However, it can either be modelled in conjunction with voluntary isolation or on its own. The adherence is estimated from reports of the 2003 SARS outbreak in Ontario to be 15% (range 5–25%) [105].

References

1. Zhang X, Meltzer MI, Wortley PM. FluSurge—a tool to estimate demand for hospital services during the next pandemic influenza. Medical Decision Making. 2006;26(6):617-23.
2. Baker P, Sun J, Morris J, Dines A. Epidemiologic modeling with FluSurge for pandemic (H1N1) 2009 outbreak, Queensland, Australia. Emerging infectious diseases. 2011;17(9):1608-14.
3. Bitar RA. Population Effects of Influenza A (H1N1) Pandemic among Health Plan Members, San Diego, California, USA, October–December 2009. Emerging infectious diseases. 2016;22(2):255.
4. Del Valle SY, Hyman JM, Hethcote HW, Eubank SG. Mixing patterns between age groups in social networks. Social Networks. 2007;29(4):539-54. doi: 10.1016/j.socnet.2007.04.005.

5. StatsCan. General Social Survey - 2010 Overview of the Time Use of Canadians. Statistics Canada, 2010.
6. Ottawa. Population and households (occupied dwellings) estimates by sub-area, year end 2014 Ottawa, ON: City Hall; 2015 [cited 2016 January 22]. Available from: http://ottawa.ca/en/city-hall/get-know-your-city/statistics/population-and-households-occupied-dwellings-estimates-sub.
7. Kelso JK, Halder N, Postma MJ, Milne GJ. Economic analysis of pandemic influenza mitigation strategies for five pandemic severity categories. BMC public health. 2013;13:211-. doi: 10.1186/1471-2458-13-211.
8. Andradottir S, Chiu W, Goldsman D, Lee ML, Tsui KL, Sander B, et al. Reactive strategies for containing developing outbreaks of pandemic influenza. BMC public health. 2011;11 Suppl 1:S1. doi: 10.1186/1471-2458-11-S1-S1. PubMed PMID: 21356128; PubMed Central PMCID: PMCPMC3317583.
9. Longini I, Halloran ME, Nizam A, Yang Y. Containing pandemic influenza with antiviral agents. American Journal of Epidemiology. 2004;159:623-33.
10. Longini I, Nizam A, Xu S, Ungchusak K, Hanshaoworakul W, Cummings DA, et al. Containing Pandemic Influenza at the source. Science. 2005;309:1083-7.
11. Chao DL, Matrajt L, Basta NE, Sugimoto JD, Dean B, Bagwell DA, et al. Planning for the control of pandemic influenza A (H1N1) in Los Angeles County and the United States. American Journal of Epidemiology. 2011;173(10):1121-30. doi: 10.1093/aje/kwq497. PubMed PMID: 21427173; PubMed Central PMCID: PMCPMC3121321.
12. Basta NE, Chao DL, Halloran ME, Matrajt L, Longini IM, Jr. Strategies for pandemic and seasonal influenza vaccination of schoolchildren in the United States. Am J Epidemiol. 2009;170(6):679-86. doi: 10.1093/aje/kwp237. PubMed PMID: 19679750; PubMed Central PMCID: PMCPMC2737588.
13. Chao DL, Halloran ME, Obenchain VJ, Longini IM, Jr. FluTE, a publicly available stochastic influenza epidemic simulation model. PLoS computational biology. 2010;6(1):e1000656. doi: 10.1371/journal.pcbi.1000656. PubMed PMID: 20126529; PubMed Central PMCID: PMCPMC2813259.
14. Germann TC, Kadau K, Longini IM, Jr., Macken CA. Mitigation strategies for pandemic influenza in the United States. Proceedings of the National Academy of Sciences USA. 2006;103(15):5935-40. doi: 10.1073/pnas.0601266103. PubMed PMID: 16585506; PubMed Central PMCID: PMCPMC1458676.
15. Del Valle SY, Hyman J, Chitnis N. Mathematical models of contact patterns between age groups for predicting the spread of infectious diseases. Mathematical Biosciences and Engineering. 2013;10(0):1475-97.
16. Bansal S, Pourbohloul B, Hupert N, Grenfell B, Meyers LA. The shifting demographic landscape of pandemic influenza. PloS one. 2010;5(2):e9360. doi: 10.1371/journal.pone.0009360. PubMed PMID: 20195468; PubMed Central PMCID: PMCPMC2829076.
17. Ross JV, House T, Keeling MJ. Calculation of disease dynamics in a population of households. PloS one. 2010;5(3):e9666. Epub 2010/03/23. doi: 10.1371/journal.pone.0009666. PubMed PMID: 20305791; PubMed Central PMCID: PMCPMC2841206.
18. Rahmandad H, Sterman J. Heterogeneity and network structure in the dynamics of diffusion: comparing agent-based and differential equation models. Management Science. 2008;54(5):998-1014.

19. Milwid R, Steriu A, Arino J, Heffernan J, Hyder A, Schanzer D, et al. Toward Standardizing a Lexicon of Infectious Disease Modeling Terms. Frontiers in Public Health. 2016;4(213). doi: 10.3389/fpubh.2016.00213.
20. Breban R, Vardavas R, Blower S. Theory versus Data: How to Calculate R(0)? PloS one. 2007;2(3):e282. doi: 10.1371/journal.pone.0000282. PubMed PMID: PMC1804098.
21. Li J, Blakeley D, Smith? RJ. The failure of R_0. Computational and mathematical methods in medicine. 2011;2011:527610. Epub 2011/08/24. doi: 10.1155/2011/527610. PubMed PMID: 21860658; PubMed Central PMCID: PMCPMC3157160.
22. Chowell G, Nishiura H, Bettencourt M. Comparative estimation of the reproduction number for pandemic influenza from daily case notification data. Journal of the Royal Society Interface. 2007;4(12):154-66.
23. van den Driessche P, Watmough J. Reproduction numbers and sub-threshold endemic equilibria for compartmental models of disease transmission. Mathematical biosciences. 2002;180:29-48. Epub 2002/10/22. PubMed PMID: 12387915.
24. Hancock K, Veguilla V, Lu X, Zhong W, Butler EN, Sun H, et al. Cross-Reactive Antibody Responses to the 2009 Pandemic H1N1 Influenza Virus. New England Journal of Medicine. 2009;361(20):1945-52. doi: 10.1056/NEJMoa0906453.
25. Mills C, Robins J, Lipsitch M. Transmissibility of 1918 pandemic influenza. Nature. 2004;432:904-6.
26. Viboud C, Tam T, Fleming D, Handel A, Miller M, Simonsen L. Transmissibility and mortality impact of epidemic and pandemic influenza, with emphasis on the unusually deadly 1951 epidemic. Vaccine. 2006;24:6701-7.
27. Coburn B, Wagner B, Blower S. Modeling Influenza epidemics and pandemics: insights into the future of swine flu (H1N1). BMC Medicine. 2009;7(30). doi: doi:10.1186/1741-7015-7-30.
28. Madhav N. Modelling a modern-day spanish flu pandemic. 2013.
29. Gojovic MZ, Sander B, Fisman D, Krahn MD, Bauch CT. Modelling mitigation strategies for pandemic (H1N1) 2009. CMAJ. 2009;181(10):673-80. doi: 10.1503/cmaj.091641. PubMed PMID: 19825923; PubMed Central PMCID: PMCPMC2774362.
30. Biggerstaff M, Cauchemez S, Reed C, Gambhir M, Finelli L. Estimates of the reproduction number for seasonal, pandemic, and zoonotic influenza: a systematic review of the literature. BMC infectious diseases. 2014;14:20. doi: 10.1186/1471-2334-14-480. PubMed PMID: WOS:000341785300001.
31. Chowell G, Ammon C, Hengartner N, Hyman J. Transmission dynamics of the great influenza pandemic of 1918 in Geneva, Switzerland: Assessing the effects of hypothetical interventions. Journal of theoretical biology. 2006;241:193-204.
32. Flahault A, Deguen S, Valleron A. A mathematical model for the European spread of influenza. European Journal of Epidemiology. 1994;10:471-4.
33. Hall I, Gani R, Hughes H, Leach S. Real-time epidemic forecasting for pandemic influenza. Epidmiology and Infection. 2007;3:372-85.
34. Sertsou G, Wilson N, Baker M, Nelson P, Roberts M. Key transmission parameters of an institutional outbreak during the 1918 influenza pandemic estimated by mathematical modelling. Theoretical Biology and Medical Modeling. 2006;3:38.
35. Basta NE, Halloran ME, Matrajt L, Longini IM, Jr. Estimating influenza vaccine efficacy from challenge and community-based study data. Am J Epidemiol. 2008;168(12):1343-52. Epub

2008/11/01. doi: 10.1093/aje/kwn259. PubMed PMID: 18974084; PubMed Central PMCID: PMCPMC2638553.
36. Conway JM, Tuite AR, Fisman DN, Hupert N, Meza R, Davoudi B, et al. Vaccination against 2009 pandemic H1N1 in a population dynamical model of Vancouver, Canada: timing is everything. BMC public health. 2011;11:932. doi: 10.1186/1471-2458-11-932. PubMed PMID: 22168242; PubMed Central PMCID: PMCPMC3280345.
37. Tuite AR, Greer AL, Whelan M, Winter AL, Lee B, Yan P, et al. Estimated epidemiologic parameters and morbidity associated with pandemic H1N1 influenza. CMAJ. 2010;182(2):131-6. doi: 10.1503/cmaj.091807. PubMed PMID: 19959592; PubMed Central PMCID: PMCPMC2817319.
38. Serres G, Rouleau I, Hamelin M, Quach C, Skowronski DM, Flamand L, et al. Contagious period for pandemic (H1N1) 2009. Emerging Infectious Diseases. 2010;16(5):783-8.
39. Arino J, Brauer F, Driessche P, Watmough J, Wu J. Simple models for containment of a pandemic. The Royal Society Interface. 2006;3:453-7. doi: 10.1098/rsif.2006.0112.
40. Arino J, Brauer F, Driessche P, Watmough J, Jianhong W. A model for influenza with vaccination and antiviral treatment. Journal of theoretical biology. 2008;253(1):118-30.
41. Lee J, Jung E. A spatial-temporal transmission model and early intervention policies of 2009 A/H1N1 influenza in South Korea. Journal of theoretical biology. 2015;380:60-73. doi: 10.1016/j.jtbi.2015.05.008. PubMed PMID: 25981631.
42. Flahault A, Vergu E, Boelle PY. Potential for a global dynamic of Influenza A (H1N1). BMC infectious diseases. 2009;9:129. doi: 10.1186/1471-2334-9-129. PubMed PMID: 19674455; PubMed Central PMCID: PMCPMC2739855.
43. Fraser C, Donnelly CA, Cauchemez S, Hanage WP, Van Kerkhove MD, Hollingsworth TD, et al. Pandemic potential of a strain of influenza A (H1N1): early findings. Science. 2009;324(5934):1557-61. doi: 10.1126/science.1176062. PubMed PMID: 19433588; PubMed Central PMCID: PMCPMC3735127.
44. Baker M, Wilson N, Huang Q. Pandemic influenza A(H1N1) in New Zealand: The experience from April to August 2009. European Surveillance. 2009;14:19319.
45. Lum M, McMillan A, Brook C, Lester R, Piers L. Impact of pandemic (H1N1) 2009 influenza on critical care capacity in Victoria. Medical Journal of Australia. 2009;191:502-6.
46. Smetanin P, Stiff D, Kumar A, Kobak P, Zarychanski M, Simonsen M, et al. Potential intensive care unit ventilator demand- capacity mismatch due to novel swine-origin H1N1 in Canada. Canadian Journal of Infectious Diseases and Medical Microbiology. 2009;20(4):e115-23.
47. CIHI. The Impact of the H1N1 Pandemic on Canadian hospitals. 2010.
48. CDC. Weekly U.S. Influenza Surveillance Report 2016 [cited 2016 October 3]. Available from: http://www.cdc.gov/flu/weekly/.
49. Kelso JK, Halder N, Milne GJ. Vaccination strategies for future influenza pandemics: a severity-based cost effectiveness analysis. BMC infectious diseases. 2013;13:81-. doi: 10.1186/1471-2334-13-81.
50. Presannis AM, Angelis D, The New York Swine Flu Investigation Team, Hagy A, Reed C, Riley S, et al. The severity of pandemic H1N1 influenza in the United States, from April to July 2009: a Bayesian analysis. PLos Medicine. 2009;6(12):e10000207.
51. Sander B, Bauch CT, Fisman D, Fowler RA, Kwong JC, Maetzel A, et al. Is a mass immunization program for pandemic (H1N1) 2009 good value for money? Evidence from the Canadian Experience. Vaccine. 2010;28(38):6210-20. doi: 10.1016/j.vaccine.2010.07.010. PubMed PMID: 20643091.

52. Hota S, Fried E, Burry L, Stewart TE, Christian MD. Preparing your intensive care unit for the second wave of H1N1 and future surges. Critical Care Medicine. 2010;38(4 Suppl):e110-9. doi: 10.1097/CCM.0b013e3181c66940. PubMed PMID: 19935417.
53. Schanzer DL, Langley JM, Tam TW. Co-morbidities associated with influenza-attributed mortality, 1994-2000, Canada. Vaccine. 2008;26(36):4697-703. doi: 10.1016/j.vaccine.2008.06.087. PubMed PMID: 18620016.
54. Jefferson T, Rivetti A, Di Pietrantonj C, Demicheli V, Ferroni E. Vaccines for preventing influenza in healthy children. Cochrane Database of Systematic Reviews. 2014;(8):N.PAG-N.PAG 1p. PubMed PMID: 105838506. Language: English. Entry Date: 20101029. Revision Date: 20150711. Publication Type: Journal Article.
55. Osterholm MT, Kelley NS, Sommer A, Belongia EA. Efficacy and effectiveness of influenza vaccines: a systematic review and meta-analysis. The Lancet Infectious Diseases. 2012;12(1):36-44. PubMed PMID: 22032844.
56. Yin JK, Chow MYK, Khandaker G, King C, Richmond P, Heron L, et al. Impacts on influenza A(H1N1)pdm09 infection from cross-protection of seasonal trivalent influenza vaccines and A(H1N1)pdm09 vaccines: Systematic review and meta-analyses. Vaccine. 2012;30(21):3209-22. PubMed PMID: 2012204311.
57. Lessler J, Reich N, Cummings DA. Outbreak of 2009 pandemic influenza A (H1N1) at a New York City school. New England Journal of Medicine. 2009;361(27):2628-36.
58. CIHI. Number of Hospital Beds Staffed and In Operation: Breakdown by grouped functional centre: Canadian MIS Database; 2016 [cited 2016 October 4]. Available from: https://www.cihi.ca/sites/default/files/document/beds_staffed_and_in_operation_2014-2015_final_en.
59. SSSQ. Institutions: Legal entities 2016 [cited 2016 October 4]. Available from: http://wpp01.msss.gouv.qc.ca/appl/M02/M02ListeEtab.asp.
60. Hill A, Fan E, Stewart T. Critical care services in Ontario: A survey-based assessment of current and future resource needs. Canadian Journal of Anaesthesiology. 2009;56:291-7.
61. Fowler RA, Abdelmalik P, Wood G, Foster D, Gibney N, Bandrauk N, et al. Critical care capacity in Canada: results of a national cross-sectional study. Crit Care. 2015;19(1):133. doi: 10.1186/s13054-015-0852-6. PubMed PMID: PMC4426537.
62. Burgess D, Jenkins G, (eds). Discount rates for the evaluation of public private partnerships. Burgess D, Jenkins G, editors. Kingston, ON: John Deutsch Institute; 2010.
63. Winquist E, Bell CM, Clarke JT, Evans G, Martin J, Sabharwal M, et al. An evaluation framework for funding drugs for rare diseases. Value in health : the journal of the International Society for Pharmacoeconomics and Outcomes Research. 2012;15(6):982-6. doi: 10.1016/j.jval.2012.06.009. PubMed PMID: 22999151.
64. Appelbaum B. As U.S. agencies put more value on a life, businesses fret. The New York Times. 2011.
65. Perlroth DJ, Glass RJ, Davey VJ, Cannon D, Garber AM, Owens DK. Health Outcomes and Costs of Community Mitigation Strategies for an Influenza Pandemic in the United States. Clinical Infectious Diseases. 2010;50(2):165-74. doi: 10.1086/649867.
66. StatsCan. Seniors 2016 [cited 2016 October 26]. Available from: http://www.statcan.gc.ca/pub/11-402-x/2011000/chap/seniors-aines/seniors-aines-eng.htm.
67. StatsCan. Earnings, average weekly, by province and territory 2016 [cited 2016 October 26]. Available from: http://www.statcan.gc.ca/tables-tableaux/sum-som/l01/cst01/labr79-eng.htm.

68. Yin JK, Khandaker G, Rashid H, Heron L, Ridda I, Booy R. Immunogenicity and safety of pandemic influenza A (H1N1) 2009 vaccine: Systematic review and meta-analysis. Influenza and other respiratory viruses. 2011;5(5):299-305. PubMed PMID: 2011443646.
69. Sander B, MecDev MBA, Nizam A, Garisson LP, Postma MJ, Halloran ME, et al. Economic Evaluation of Influenza Pandemic Mitigation Strategies in the United States Using a Stochastic Microsimulation transmission model. Value in Health. 2009;12. doi: 10.1111/j.1524-4733.2008.00437.x.
70. PHAC. Vaccine coverage amongst adult Canadians: Results from the 2012 adult National Immunization Coverage (aNIC) survey 2014 [cited 2016 November 7]. Available from: http://www.phac-aspc.gc.ca/im/nics-enva/vcac-cvac-eng.php.
71. Li ZY, Chen JY, Zhang YL, Fu WM. Partial protection against 2009 pandemic influenza A (H1N1) of seasonal influenza vaccination and related regional factors: Updated systematic review and meta-analyses. Human Vaccines Immunother. 2015;11(6):1337-44. PubMed PMID: 25692308; PubMed Central PMCID: PMCPMC4514212.
72. Jefferies S, Earl D, Berry N, Blackmore T, Rooker S, Raymond N, et al. Effectiveness of the 2009 seasonal influenza vaccine against pandemic influenza A(H1N1)2009 in healthcare workers in New Zealand, June-August 2009. Euro Surveillance. 2011;16(2).
73. Hardelid P, Fleming D, McMenamin J, Andrews N, Robertson C, SebastianPillai P, et al. Effectiveness of pandemic and seasonal influenza vaccine in preventing pandemic influenza A(H1N1)2009 infection in England and Scotland 2009-2010. Euro Surveillance. 2011;16(2).
74. Kelly H, Grant K. Interim analysis of pandemic influenza (H1N1) 2009 in Australia: Surveillance trends, age of infection and effectiveness of seasonal vaccination. Euro Surveillance. 2009;14(31).
75. Mercer GN, Barry SI, Kelly H. Modelling the effect of seasonal influenza vaccination on the risk of pandemic influenza infection. BMC public health. 2011;11(1):S11. doi: 10.1186/1471-2458-11-s1-s11.
76. Saunders-Hastings P, Reisman J, Krewski D. Assessing the State of Knowledge Regarding the Effectiveness of Interventions to Contain Pandemic Influenza Transmission: A Systematic Review and Narrative Synthesis. PloS one. 2016;11(12):e0168262. doi: 10.1371/journal.pone.0168262.
77. Fiore A, Uyeki T, Broder K, Finelli L, Euler GL, Singleton JA, et al. Prevention and Control of Influenza with Vaccines Recommendations of the Advisory Committee on Immunization Practices (ACIP), 2010. Morbidity and Mortality Weekly Report. 2010;59:1-64.
78. Khazeni N, Bravata DM, Holty JE, Uyeki TM, Stave CD, Gould MK. Systematic review: safety and efficacy of extended-duration antiviral chemoprophylaxis against pandemic and seasonal influenza. Annals of internal medicine. 2009;151(7):464-73. Epub 2009/08/05. PubMed PMID: 19652173.
79. Jefferson T, Jones MA, Doshi P, Del Mar CB, Hama R, Thompson MJ, et al. Neuraminidase inhibitors for preventing and treating influenza in healthy adults and children. The Cochrane database of systematic reviews. 2014;4:CD008965. doi: 10.1002/14651858.CD008965.pub4. PubMed PMID: 24718923.
80. Araz OM, Damien P, Paltiel DA, Burke S, van de Geijn B, Galvani A, et al. Simulating school closure policies for cost effective pandemic decision making. BMC public health. 2012;12(1):1-11. doi: 10.1186/1471-2458-12-449.

81. Halder N, Kelso JK, Milne GJ. Analysis of the effectiveness of interventions used during the 2009 A/H1N1 influenza pandemic. BMC public health. 2010;10(1):168. doi: 10.1186/1471-2458-10-168.
82. Wong ZS-Y, Goldsman D, Tsui K-L. Economic Evaluation of Individual School Closure Strategies: The Hong Kong 2009 H1N1 Pandemic. PloS one. 2016;11(1):e0147052. doi: 10.1371/journal.pone.0147052. PubMed PMID: PMC4731466.
83. Borse RH, Behravesh CB, Dumanovsky T, Zucker JR, Swerdlow D, Edelson P, et al. Closing schools in response to the 2009 pandemic influenza A H1N1 virus in New York City: economic impact on households. Clinical infectious diseases : an official publication of the Infectious Diseases Society of America. 2011;52 Suppl 1:S168-72. doi: 10.1093/cid/ciq033. PubMed PMID: 21342890.
84. Zayas G, Chiang MC, Wong E, MacDonald F, Lange CF, Senthilselvan A, et al. Effectiveness of cough etiquette maneuvers in disrupting the chain of transmission of infectious respiratory diseases. BMC public health. 2013;13(1):1-11. doi: 10.1186/1471-2458-13-811.
85. Zayas G, Chiang MC, Wong E, MacDonald F, Lange CF, Senthilselvan A, et al. Cough aerosol in healthy participants: Fundamental knowledge to optimize droplet-spread infectious respiratory disease management. BMC Pulmonary Medicine. 2012;12:no pagination.
86. Zayas JG, Chiang MC, Wong E, MacDonald F, Lange C, Sentilselvan A, et al. Improving the quality of interventions to control infectious cough aerosol droplets. American Journal of Respiratory and Critical Care Medicine. 2012;185:no pagination.
87. Zayas JG, Chiang MJ, Wong EYL, MacDonald F, Lange C, Senthilselvan A, et al. Human cough model and bioaerosol characterization in influenza pandemic containment. American Journal of Respiratory and Critical Care Medicine. 2010;181(1 MeetingAbstracts):no pagination.
88. Godoy P, Castilla J, Delgado-Rodriguez M, Martin V, Soldevila N, Alonso J, et al. Effectiveness of hand hygiene and provision of information in preventing influenza cases requiring hospitalization. Preventive medicine. 2012;54(6):434-9. Epub 05/03. doi: 10.1016/j.ypmed.2012.04.009. PubMed PMID: 22548868.
89. Li T, Liu Y, Di B, Wang M, Shen J, Zhang Y, et al. Epidemiological investigation of an outbreak of pandemic influenza A (H1N1) 2009 in a boarding school: Serological analysis of 1570 cases. Journal of Clinical Virology. 2011;50(3):235-9.
90. Liu WT, Pang XH, Deng Y, Yang P, Li XY, Zhang Y, et al. [A case-control study of the transmission of pandemic influenza A (H1N1) virus in families]. Zhonghua jie he he hu xi za zhi = Zhonghua jiehe he huxi zazhi = Chinese journal of tuberculosis and respiratory diseases. 2011;34(7):509-14.
91. Torner N, Soldevila N, Garcia JJ, Launes C, Godoy P, Castilla J, et al. Effectiveness of non-pharmaceutical measures in preventing pediatric influenza: a case-control study. BMC public health. 2015;15:543. Epub 06/10. doi: 10.1186/s12889-015-1890-3. PubMed PMID: 26055522.
92. Zhang Y, Seale H, Yang P, MacIntyre CR, Blackwell B, Tang S, et al. Factors associated with the transmission of pandemic (H1N1) 2009 among hospital healthcare workers in Beijing, China. Influenza and other respiratory viruses. 2012;7(3):466-71. Epub 10/20. doi: 10.1111/irv.12025. PubMed PMID: 23078163.
93. Cheng VCC, Tai JWM, Wong LMW, Chan JFW, Li IWS, To KKW, et al. Prevention of nosocomial transmission of swine-origin pandemic influenza virus A/H1N1 by infection control bundle. The Journal of hospital infection. 2010;74(3):271-7.

94. Deng Y, Zhang Y, Wang XL, Liu WT, Duan W, Yang P, et al. [Pandemic influenza A (H1N1) virus infection factors among healthcare workers - a case-control study]. Zhonghua Yu Fang Yi Xue Za Zhi. 2010;44(12):1075-8. Epub 2011/01/11. PubMed PMID: 21215106.
95. Jaeger JL, Patel M, Dharan N, Hancock K, Meites E, Mattson C, et al. Transmission of 2009 Pandemic Influenza A (H1N1) Virus among Healthcare Personnel-Southern California, 2009. Infection Control & Hospital Epidemiology. 2011;32(12):1149-57 9p. PubMed PMID: 108203657. Language: English. Entry Date: 20120127. Revision Date: 20150818. Publication Type: Journal Article.
96. Remschmidt C, Stocker P, Heiden Mad, Suess T, Luchtenberg M, Schink SB, et al. Preventable and non-preventable risk factors for influenza transmission and hygiene behavior in German influenza households, pandemic season (H1N1) 2009/2010. Influenza and other respiratory viruses. 2013;7(3):418-25.
97. Suess T, Remschmidt C, Schink SB, Schweiger B, Nitsche A, Schroeder K, et al. The role of facemasks and hand hygiene in the prevention of influenza transmission in households: results from a cluster randomised trial; Berlin, Germany, 2009-2011. BMC infectious diseases. 2012;12:26. Epub 01/28. doi: 10.1186/1471-2334-12-26. PubMed PMID: 22280120.
98. Merk H, Kuhlmann-Berenzon S, Linde A, Nyren O. Associations of hand-washing frequency with incidence of acute respiratory tract infection and influenza-like illness in adults: a population-based study in Sweden. BMC infectious diseases. 2014;14:509. Epub 2014/09/23. doi: 10.1186/1471-2334-14-509. PubMed PMID: 25234544; PubMed Central PMCID: PMCPMC4177698.
99. Tooher R, Collins JE, Street JM, Braunack-Mayer A, Marshall H. Community knowledge, behaviours and attitudes about the 2009 H1N1 Influenza pandemic: a systematic review. Influenza and other respiratory viruses. 2013;7(6):1316-27. doi: 10.1111/irv.12103. PubMed PMID: WOS:000331001400059.
100. Seale H, Heywood AE, McLaws M-L, Ward KF, Lowbridge CP, Van D, et al. Why do I need it? I am not at risk! Public perceptions towards the pandemic (H1N1) 2009 vaccine. BMC infectious diseases. 2010;10(1):99. doi: 10.1186/1471-2334-10-99.
101. Kim CO, Nam CM, Lee DC, Chang J, Lee JW. Is abdominal obesity associated with the 2009 influenza A (H1N1) pandemic in Korean school-aged children? Influenza and other respiratory viruses. 2012;6(5):313-7.
102. Bults M, Beaujean DJ, de Zwart O, Kok G, van Empelen P, van Steenbergen JE, et al. Perceived risk, anxiety, and behavioural responses of the general public during the early phase of the Influenza A (H1N1) pandemic in the Netherlands: results of three consecutive online surveys. BMC public health. 2011;11(1):2. doi: 10.1186/1471-2458-11-2.
103. Haber MJ, Shay D, K., Davis XM, Patel R, Jin X, Weintraub E. Effectiveness of interventions to reduce contact rates during a simulated influenza pandemic. Emerging Infectious Diseases. 2007;13(4). doi: 10.3201/eid1304.060828.
104. Zhang Q, Wang D. Assessing the Role of Voluntary Self-Isolation in the Control of Pandemic Influenza Using a Household Epidemic Model. International Journal of Environmental Research and Public Health. 2015;12(8):9750-67. doi: 10.3390/ijerph120809750. PubMed PMID: PMC4555310.
105. Bondy SJ, Russell ML, Lafleche JM, Rea E. Quantifying the impact of community quarantine on SARS transmission in Ontario: estimation of secondary case count difference and number needed to quarantine. BMC public health. 2009;9:488. doi: 10.1186/1471-2458-9-488. PubMed PMID: 20034405; PubMed Central PMCID: PMCPMC2808319.
