# Supplementary material for: Modelling community-control strategies to protect hospital resources during an influenza pandemic in Ottawa, Canada
Source: PLoS One. 2017 Jun 14;12(6):e0179315. doi: 10.1371/journal.pone.0179315 (PMC5470707; doi:10.1371/journal.pone.0179315)
Supplement: S1 Table — (PDF) [file pone.0179315.s002.pdf]

S1 Table. Ottawa–Gatineau CMA profile

Table S1.1 outlines the parameter values of interest for the study population.

Table S1.1. Ottawa–Gatineau CMA profile.

**Population By Age**	
0–4	71,245
5–18	204,920
19–29	190,640
30–64	622,640
65+	156,875
Total	1,246,320
**Average Earnings/Employment**	
Earnings Per Week (CAD)	$962.73
Earnings Per Day (CAD)	$192.55
Unemployment Rate (%)	6.5
**Hospital Resources**	
Acute Beds	1,822
ICU beds	269
Other Beds	2,245
Total Beds	4,336
