# Supplementary material for: Modelling community-control strategies to protect hospital resources during an influenza pandemic in Ottawa, Canada
Source: PLoS One. 2017 Jun 14;12(6):e0179315. doi: 10.1371/journal.pone.0179315 (PMC5470707; doi:10.1371/journal.pone.0179315)
Supplement: S2 Table — (PDF) [file pone.0179315.s003.pdf]

S2 Table. Results of Basic Analysis: Symptomatic Infection

Table S2.1 provides the best-guess results for the number of symptomatic infections predicted for each of the 192 intervention bundles.

Table S2.1. **Predicted number of symptomatic infections (95% confidence intervals)**

**Non-pharmaceutical intervention component**	**Pharmaceutical intervention component**							
	**None**	**V**	**AVT**	**AVP**	**V+AVT**	**V+AVP**	**AVT+AVP**	**V+AVT+AVP**
**None**	677,545.6 (675,672.7-679,418.5)	624,361.0 (622,704.8-626,017.1)	676,264.3 (674,397.7-678,130.9)	658,482.4 (656,726.4-660,238.4)	622,681.4 (621,032.4-624,330.4)	602,439.4 (600,895.8-603,983.0)	656,784.5 (655,036.0-658,533.1)	600,393.7 (598,857.7-601,929.6)
**SC**	669,926.3 (668,396.9-671,455.8)	615,810.6 (614,462.5-617,158.8)	668,568.7 (557,044.7-670,092.7)	650,145.8 (648,706.4-651,585.2)	614,039.0 (612,696.9-615,391.0)	593,213.0 (591,951.1-594,474.9)	648,352.9 (646,919.8-649,785.9)	591,062.2 (589,806.9-592,317.6)
**CCR**	673,285.1 (671,438.9-675,131.4)	618,840.9 (617,212.0-620,132.4)	671,954.8 (670,114.9-673,794.7)	653,202.5 (651,473.8-654,931.1)	617,095.2 (615,473.5-618,717.0)	595,677.8 (594,161.9-597,193.7)	651,434.2 (649,713.0-653,155.4)	593,541.2 (592,032.9-595,049.6)
**PPM**	566,469.3 (564,972.2-567,966.3)	481,604.2 (480,337.0-482,871.3)	563,665.9 (562,174.7-565,157.1)	517,570.0 (516,202.4-518,937.6)	477,916.2 (476,656.2-479,176.2)	427,382.3 (426,248.4-428,516.2)	513,693.6 (512,333.3-515,053.8)	422,936.1 (421,810.9-424,061.3)
**VI**	429,995.3 (428,933.3-431,057.3)	361,321.5 (360,432.4-362,210.6)	427,631.6 (426,579.9-428,689.2)	398,100.5 (397,117.1-399,083.9)	359,612.4 (358,728.8-360,496.1)	327,559.5 (326,750.9-328,368.1)	395,034.3 (394,056.5-396,012.2)	324,237.1 (323,434.8-325,039.4)
**Q**	422,404.3 (421,358.8-423,449.9)	352,957.7 (352,086.3-353,829.1)	419,983.8 (418,941.7-421,024.9)	389,727.7 (388,761.6-396,693.7)	351,256.3 (350,390.3-352,122.3)	318,687.9 (317,898.1-319,477.7)	386,599.7 (385,639.3-387,560.1)	315,327.6 (314,544.2-316,111.0)
**SC+CCR**	664,585.0 (663,081.3-666,088.7)	608,993.0 (607,670.7-610,315.2)	663,171.3 (661,673.1-664,669.5)	643,580.7 (642,167.4-644,994.0)	607,140.6 (605,824.4-608,456.8)	585,006.4 (583,770.7-586,242.1)	641,703.0 (640,296.1-643,109.9)	582,748.2 (581,519.0-583,977.4)
**SC+PPM**	551,833.9 (550,620.1-553,047.6)	466,171.1 (465,151.2-467,191.1)	548,868.3 (547,659.6-550,076.9)	502,294.3 (501,183.0-503,405.5)	462,338.3 (461,324.7-463,351.9)	412,295.5 (411,382.3-413,208.8)	498,262.3 (497,157.6-499,367.0)	408,108.7 (407,2020.8-409,014.7)

**SC+VI**	404,037.0 (403,239.7-404,834.4)	340,858.5 (340,192.0-341,524.9)	401,751.4 (400,957.6-402,545.2)	377,595.9 (376,848.2-378,343.7)	339,254.4 (338,592.1-339,916.6)	313,099.3 (312,483.3-313,715.3)	374,688.0 (373,944.7-375,431.3)	309,995.2 (309,384.1-310,606.3)
**SC+Q**	397,015.4 (396,229.6-397,801.3)	333,341.0 (332,686.7-333,995.3)	394,691.0 (393,908.7-395,473.3)	370,017.0 (369,281.5-370,752.6)	331,784.6 (331,098.5-332,398.8)	305,329.2 (304,725.9-305,932.4)	367,067.3 (366,336.2-367,798.3)	302,193.4 (301,595.0-302,791.7)
**CCR+PPM**	554,925.6 (553,450.3-556,400.9)	467,353.0 (466,111.1-468,594.8)	551,950.3 (550,480.8-553,419.7)	503,121.8 (501,779.4-504,464.3)	463,477.3 (462,242.9-464,711.7)	410,710.8 (409,607.5-411,814.1)	499,033.5 (497,698.7-500,368.3)	406,098.0 (405,003.8-407,192.1)
**CCR+VI**	418,722.9 (417,684.0-419,761.7)	348,947.7 (348,083.6-349,811.7)	416,265.3 (415,230.9-417,299.6)	384,181.9 (383,223.5-385,140.3)	345,958.0 (345,099.5-346,816.4)	313,135.0 (312,354.4-313,915.6)	381,359.4 (380,406.8-382,311.9)	309,735.0 (308,961.0-310,509.1)
**CCR+Q**	414,791.9 (413,761.5-415,822.4)	344,659.4 (343,804.4-345,514.4)	412,305.8 (411,279.8-413,331.7)	379,839.2 (378,889.7-380,788.6)	341,645.6 (340,796.2-342,494.9)	308,614.1 (307,843.2-309,385.0)	377,003.0 (376,059.4-377,946.6)	305,198.5 (304,434.3-305,962.8)
**PPM+VI**	219,412.5 (218,760.7-220,064.2)	155,234.1 (154,784.2-155,684.0)	216,115.5 (215,472.0-216,758.9)	176,698.9 (176,177.4-177,220.5)	152,190.3 (151,748.7-152,631.9)	122,052.8 (121,707.6-122,398.1)	173,209.5 (172,697.4-173,721.6)	119,291.8 (118,954.4-119,629.3)
**PPM+Q**	209,894.0 (209,268.9-210,519.1)	147,486.9 (147,060.2-147,913.7)	206,643.7 (206,027.0-207,136.1)	168,239.4 (167,763.0-168,735.8)	144,530.3 (144,111.8-144,948.9)	115,608.6 (115,283.2-115,934.1)	164,838.6 (164,351.6-165,325.7)	112,951.4 (112,633.5-113,269.3)
**SC+CCR+PPM**	538,366.3 (537,174.5-539,558.1)	450,131.1 (449,136.5-451,125.7)	535,216.1 (534,029.5-536,402.7)	485,995.2 (484,909.2-487,081.1)	446,110.6 (445,122.6-447,098.6)	394,095.3 (393,211.9-394,978.6)	481,748.6 (480,669.5-482,827.7)	89,405.9 (388,530.6-390,281.1)
**SC+CCR+VI**	392,272.7 (391,494.9-393,050.6)	328,574.7 (327,928.5-329,220.9)	390,043.7 (389,369.7-390,817.6)	363,723.5 (362,996.5-364,450.4)	325,769.8 (325,128.0-326,411.7)	299,054.2 (298,460.2-299,648.1)	361,060.5 (360,338.2-361,782.8)	295,888.4 (295,299.4-296,477.3)
**SC+CCR+Q**	388,785.1 (388,013.4-389,556.8)	324,732.6 (324,092.7-325,372.6)	386,403.6 (385,635.5-387,171.7)	359,808.0 (359,087.4-360,528.7)	321,912.9 (321,277.3-322,548.6)	295,038.2 (294,450.9-295,625.5)	357,138.6 (356,422.6-357,854.6)	291,863.0 (291,280.7-292,445.3)
**SC+PPM+VI**	207,908.3 (207,424.6-208,392.0)	149,873.1 (149,530.9-150,215.4)	204,853.9 (204,376.1-205,331.7)	171,437.2 (171,036.7-171,837.8)	147,034.9 (146,698.8-147,371.1)	120,501.3 (120,229.3-120,773.3)	168,134.1 (167,740.5-168,527.7)	117,821.1 (117,555.0-118,087.1)

**SC+PPM+Q**	199,537.5 (199,070.8-200,004.2)	142,904.2 (142,577.2-143,231.1)	196,550.5 (196,089.9-197,011.1)	163,795.8 (163,412.0-164,179.6)	140,100.7 (139,779.7-140,421.7)	114,484.0 (114,225.7-114,742.2)	160,563.8 (160,186.9-160,940.7)	111,893.9 (111,641.5-112,146.4)
**CCR+PPM+VI**	203,770.7 (203,160.2-204,381.2)	141,972.4 (141,559.7-142,385.1)	200,536.9 (199,934.9-201,138.9)	161,963.3 (161,482.9-162,443.6)	139,066.3 (138,661.7-139,470.9)	110,768.1 (110,455.5-111,080.6)	157,867.3 (157,397.9-158,336.7)	108,178.0 (107,872.8-108,483.1)
**CCR+PPM+Q**	199,101.2 (198,504.2-199,698.3)	138,272.4 (137,871.0-138,673.9)	195,896.8 (195,308.3-196,485.4)	157,905.4 (157,437.4-158,373.3)	135,413.1 (135,019.7-135,806.5)	107,729.2 (107,426.1-108,032.3)	153,868.1 (153,411.0-154,325.2)	105,191.9 (104,896.1-105,487.7)
**SC+CCR+PPM+VI**	193,101.9 (192,647.5-193,556.4)	137,165.9 (136,850.3-137,481.4)	190,106.0 (189,657.6-190,554.4)	157,202.8 (156,832.2-157,573.3)	134,411.7 (134,102.1-134,721.2)	109,358.7 (109,111.2-109,606.1)	153,353.4 (152,990.8-153,715.9)	106,856.3 (106,614.6-107,098.1)
**SC+CCR+PPM+Q**	189,014.7 (188,568.8-189,460.7)	133,807.2 (133,499.1-134,115.2)	186,025.1 (185,585.3-186,465.0)	153,539.8 (153,177.6-153,902.1)	131,093.3 (130,791.1-131,395.5)	106,520.4 (106,279.6-106,761.2)	149,706.7 (149,352.4-150,060.9)	104,050.6 (103,815.4-104,285.8)
