# Supplementary material for: Modelling community-control strategies to protect hospital resources during an influenza pandemic in Ottawa, Canada
Source: PLoS One. 2017 Jun 14;12(6):e0179315. doi: 10.1371/journal.pone.0179315 (PMC5470707; doi:10.1371/journal.pone.0179315)
Supplement: S3 Table — (PDF) [file pone.0179315.s004.pdf]

# S3 Table. Results of Basic Analysis: Acute Care Hospital Admissions

**Table S3.1** provides the best-guess results for the number of hospitalizations predicted for each of the 192 intervention bundles.

**Table S3.1. Predicted number of hospitalizations (95% confidence intervals)**

| Non-pharmaceutical intervention component | Pharmaceutical intervention component | | | | | | | |
|---|---|---|---|---|---|---|---|---|
| | **None** | **V** | **AVT** | **AVP** | **V+AVT** | **V+AVP** | **AVT+AVP** | **V+AVT+AVP** |
| **None** | 2,472.0 (2,467.7-2,476.3) | 909.7 (908.2-911.3) | 2,218.6 (2,214.7-2,222.4) | 2,110.8 (2,107.2-2,114.3) | 815.4 (814.0-816.7) | 769.5 (768.2-770.7) | 2,101.7 (2,098.2-2,105.3) | 765.4 (764.2-766.7) |
| **SC** | 2,430.1 (2,426.5-2,433.6) | 890.9 (889.6-892.1) | 2,180.5 (2,177.3-2,183.7) | 2,069.7 (2,066.7-2,072.7) | 798.2 (797.1-799.4) | 751.4 (750.3-752.4) | 2,060.3 (2,057.3-2,063.2) | 747.2 (746.2-748.2) |
| **CCR** | 2,452.3 (2,448.1-2,456.6) | 899.7 (898.2-901.2) | 2,200.7 (2,196.9-2,204.5) | 2,089.0 (2,085.5-2,092.6) | 806.3 (804.9-807.6) | 758.6 (757.4-759.8) | 2,079.8 (2,076.3-2,083.3) | 745.5 (753.2-755.7) |
| **PPM** | 1,956.7 (1,953.2-1,960.2) | 655.8 (653.6-657.9) | 1,749.4 (1,746.2-1,752.5) | 1,550.0 (1,547.2-1,552.8) | 584.3 (583.2-585.3) | 504.0 (503.1-504.9) | 1,534.3 (1,531.5-1,537.1) | 497.4 (496.5-498.4) |
| **VI** | 1,479.9 (1,477.6-1,482.1) | 492.0 (491.3-492.8) | 1,322.5 (1,320.5-1,324.6) | 1,194.0 (1,192.3-1,195.8) | 440.3 (439.6-440.9) | 388.6 (388.0-389.2) | 1,182.0 (1,180.3-1,183.8) | 383.7 (383.2-384.3) |
| **Q** | 1,448.9 (1,447.3-1,450.5) | 478.9 (478.4-479.5) | 1,294.5 (1,293.1-1,296.0) | 1,164.8 (1,163.6-1,166.0) | 428.5 (428.0-429.0) | 376.7 (376.3-377.1) | 1,152.7 (1,151.5-1,153.9) | 371.9 (371.5-372.3) |
| **SC+CCR** | 2,405.9 (2,402.4-2,409.4) | 878.7 (877.4-879.9) | 2,158.6 (2,155.4-2,161.7) | 2,043.2 (2,040.3-2,046.1) | 787.1 (786.0-788.3) | 739.4 (737.4-739.5) | 2,033.5 (2,030.6-2,036.4) | 734.1 (733.1-735.1) |
| **SC+PPM** | 1,881.2 (1878.3-1,884.1) | 625.3 (624.3-626.3) | 1,680.9 (1,678.3-1,683.5) | 1,482.8 (1,480.5-1,485.0) | 556.7 (555.9-557.6) | 478.6 (477.9-479.4) | 1,466.8 (1,464.5-1,469.0) | 472.6 (471.8-473.3) |

| | | | | | | | | |
|---|---|---|---|---|---|---|---|---|
| **SC+VI** | 1,338.2 (1,336.5-1,339.9) | 444.1 (443.5-444.6) | 1,195.4 (1,193.8-1,196.9) | 1,087.9 (1,086.6-1,089.2) | 397.3 (396.8-367.8) | 354.6 (354.2-355.0) | 1,076.6 (1,075.3-1,077.9) | 350.1 (349.7-350.5) |
| **SC+Q** | 1,309.9 (1,308.7-1,311.1) | 432.5 (432.1-432.8) | 1,169.8 (1,168.8-1,170.9) | 1,061.8 (1,060.8-1,062.7) | 386.9 (386.5-387.2) | 344.6 (344.3-344.9) | 1,050.4 (1,049.4-1,051.3) | 340.1 (339.8-340.4) |
| **CCR+PPM** | 1,905.0 (1,901.6-1,908.4) | 631.9 (630.8-633.1) | 1,702.3 (1,699.2-1,705.4) | 1,496.2 (1,493.5-1,498.9) | 562.6 (561.6-563.7) | 480.9 (480.0-481.8) | 1,480.0 (1,477.3-1,482.8) | 474.2 (473.3-475.1) |
| **CCR+VI** | 1,433.6 (1,431.4-1,435.9) | 378.1 (377.5-378.6) | 472.6 (471.8-473.3) | 1,144.7 (1,143.0-1,146.4) | 420.8 (420.1-421.4) | 369.1 (368.6-369.7) | 1,133.9 (1,132.2-1,135.6) | 364.2 (363.7-364.8) |
| **CCR+Q** | 1,417.7 (1,416.2-1,419.3) | 465.9 (465.4-466.4) | 1,266.3 (1,264.9-1,267.7) | 1,129.7 (1,128.5-1,130.9) | 414.8 (414.3-415.2) | 363.1 (362.8-363.5) | 1,118.9 (1,117.7-1,120.1) | 358.3 (357.9-358.7) |
| **PPM+VI** | 686.5 (685.2-687.7) | 194.3 (193.9-194.6) | 607.6 (606.5-608.7) | 484.9 (484.1-485.8) | 171.2 (170.9-171.5) | 134.7 (134.5-135.0) | 474.5 (473.7-475.3) | 131.5 (131.3-131.7) |
| **PPM+Q** | 654.6 (653.8-655.5) | 184.2 (184.0-184.5) | 579.2 (578.4-579.9) | 460.8 (460.2-461.3) | 162.3 (162.1-162.5) | 127.5 (127.4-127.7) | 450.6 (450.1-451.2) | 124.4 (124.3-124.6) |
| **SC+CCR+PPM** | 1,822.7 (1,819.9-1,825.6) | 599.3 (598.3-600.2) | 1,627.8 (1,625.2-1,630.3) | 1,423.9 (1,421.7-1,426.1) | 533.1 (532.3-534.0) | 454.1 (453.4-454.8) | 1,407.5 (1,405.3-1,409.7) | 447.4 (446.7-448.1) |
| **SC+CCR+VI** | 1,290.8 (1,289.1-1,292.4) | 425.3 (424.7-425.8) | 1,153.5 (1,152.0-1,154.9) | 1,039.9 (1,038.6-1,041.2) | 378.5 (378.0-379.0) | 336.1 (335.7-336.5) | 1,029.7 (1,028.5-1,031.0) | 331.6 (331.2-332.0) |
| **SC+CCR+Q** | 1,277.4 (1,276.2-1,278.5) | 419.4 (419.0-419.8) | 1,140.4 (1,139.4-1,141.5) | 1,026.5 (1,025.6-1,027.4) | 373.2 (372.9-373.6) | 330.9 (330.6-331.2) | 1,016.4 (1,015.5-1,017.3) | 326.4 (326.1-326.7) |
| **SC+PPM+VI** | 610.0 (609.1-610.8) | 175.2 (175.0-175.5) | 539.8 (539.0-540.5) | 441.3 (440.7-441.9) | 154.7 (154.5-154.9) | 124.8 (124.7-125.0) | 431.7 (431.2-432.2) | 121.9 (121.7-122.0) |

| | | | | | | | | |
|---|---|---|---|---|---|---|---|---|
| **SC+PPM+Q** | 583.0 (582.4-583.6) | 166.9 (166.8-167.1) | 516.5 (516.0-517.1) | 421.1 (420.6-421.5) | 147.0 (146.9-147.2) | 118.7 (118.6-118.8) | 411.8 (411.4-412.2) | 115.8 (115.7-115.9) |
| **CCR+PPM+VI** | 633.6 (632.5-634.8) | 176.9 (176.6-177.2) | 560.4 (599.4-561.4) | 442.4 (441.7-443.2) | 155.7 (155.5-156.0) | 122.0 (121.8-122.2) | 430.3 (429.6-431.0) | 119.0 (118.8-119.2) |
| **CCR+PPM+Q** | 618.2 (617.4-619.0) | 172.2 (172.0-172.4) | 546.7 (546.0-547.4) | 430.9 (430.4-431.5) | 151.5 (151.3-151.7) | 118.6 (118.5-118.7) | 419.0 (418.5-419.5) | 115.7 (115.5-115.8) |
| **SC+CCR+PPM+VI** | 562.5 (561.7-563.3) | 159.9 (159.7-160.1) | 497.4 (496.7-498.1) | 402.5 (402.0-403.1) | 140.8 (140.6-141.0) | 113.1 (112.9-113.2) | 392.2 (391.7-392.7) | 110.5 (110.4-110.7) |
| **SC+CCR+PPM+Q** | 549.5 (548.9-550.1) | 155.8 (155.7-156.0) | 485.8 (485.3-486.3) | 393.3 (392.9-393.7) | 137.2 (137.0-137.3) | 110.3 (110.2-110.4) | 382.4 (382.1-382.8) | 107.6 (107.5-107.7) |
