# Supplementary material for: Modelling community-control strategies to protect hospital resources during an influenza pandemic in Ottawa, Canada
Source: PLoS One. 2017 Jun 14;12(6):e0179315. doi: 10.1371/journal.pone.0179315 (PMC5470707; doi:10.1371/journal.pone.0179315)
Supplement: S4 Table — (PDF) [file pone.0179315.s005.pdf]

S4 Table. Results of Basic Analysis: Peak Acute Care Hospitalization Demand

Table S4.1 provides the best-guess results for the peak acute care hospital demand (as a percentage of all acute hospital beds in the Ottawa–Gatineau CMA) for each of the 192 intervention bundles.

Table S4.1. Predicted peak acute care hospital bed demand (95% confidence intervals)

Non-pharmaceutical intervention component	Pharmaceutical intervention component							
	None	V	AVT	AVP	V+AVT	V+AVP	AVT+AVP	V+AVT+AVP
**None**	13.8 (13.1-14.5)	4.9 (4.6-5.1)	12.4 (11.7-13.0)	11.4 (10.8-11.9)	4.4 (4.1-4.6)	4.0 (3.8-4.2)	11.3 (10.7-11.9)	4.0 (3.8-4.2)
**SC**	13.3 (12.6-14.0)	4.7 (4.5-4.9)	11.9 (11.3-12.5)	10.9 (10.4-11.5)	4.2 (4.0-4.4)	3.8 (3.6-4.0)	10.9 (10.3-11.4)	3.8 (3.6-4.0)
**CCR**	13.6 (12.9-14.3)	4.8 (4.5-5.0)	12.2 (11.6-12.8)	11.2 (10.6-11.7)	4.3 (4.1-4.5)	3.9 (3.7-4.1)	11.1 (10.6-11.7)	3.9 (3.7-4.1)
**PPM**	10.5 (10.0-11.0)	3.5 (3.3-3.7)	9.4 (8.9-9.9)	8.3 (7.9-8.7)	3.1 (3.0-3.3)	2.7 (2.6-2.8)	8.2 (7.8-8.6)	2.7 (2.6-2.8)
**VI**	0.7 (0.7-0.8)	0.2 (0.2-0.2)	0.6 (0.6-0.7)	0.8 (0.8-0.9)	0.2 (0.2-0.2)	0.3 (0.3-0.3)	0.8 (0.8-0.9)	0.3 (0.3-0.3)
**Q**	0.7 (0.6-0.7)	0.2 (0.2-0.2)	0.6 (0.6-0.6)	0.8 (0.8-0.9)	0.2 (0.2-0.2)	0.3 (0.3-0.3)	0.8 (0.8-0.9)	0.3 (0.3-0.3)
**SC+CCR**	13.1 (12.4-13.7)	4.6 (4.4-4.8)	11.7 (11.1-12.3)	10.7 (10.2-11.3)	4.1 (3.9-4.3)	3.7 (3.5-3.9)	10.7 (10.1-11.2)	3.7 (3.5-3.9)
**SC+PPM**	10.0 (9.5-10.5)	3.3 (3.2-3.5)	9.0 (8.5-9.4)	7.9 (7.5-8.3)	3.0 (2.8-3.1)	2.6 (2.4-2.7)	7.8 (7.5-8.2)	2.6 (2.4-2.7)

**SC+VI**	1.2 (1.1-1.3)	0.4 (0.4-0.4)	1.1 (1.0-1.1)	1.2 (1.1-1.3)	0.4 (0.3-0.4)	0.4 (0.4-0.4)	1.2 (1.1-1.3)	0.4 (0.4-0.4)
**SC+Q**	1.2 (1.1-1.2)	0.4 (0.4-0.4)	1.0 (1.0-1.1)	1.2 (1.1-1.2)	0.3 (0.3-0.4)	0.4 (0.4-0.4)	1.2 (1.1-1.2)	0.4 (0.4-0.4)
**CCR+PPM**	10.3 (9.8-10.8)	3.4 (3.2-3.6)	9.2 (8.8-9.7)	8.1 (7.7-8.5)	3.1 (2.9-3.2)	2.6 (2.5-2.8)	8.0 (7.6-8.4)	2.6 (2.5-2.7)
**CCR+VI**	0.7 (0.6-0.7)	0.2 (0.2-0.2)	0.2 (0.2-0.2)	0.8 (0.8-0.9)	0.2 (0.2-0.2)	0.3 (0.3-0.3)	0.8 (0.8-0.9)	0.3 (0.3-0.3)
**CCR+Q**	0.7 (0.6-0.7)	0.2 (0.2-0.2)	0.6 (0.6-0.6)	0.8 (0.8-0.9)	0.2 (0.2-0.2)	0.3 (0.3-0.3)	0.8 (0.8-0.9)	0.3 (0.3-0.3)
**PPM+VI**	0.5 (0.4-0.5)	0.1 (0.1-0.1)	0.4 (0.4-0.4)	0.5 (0.5-0.6)	0.1 (0.1-0.1)	0.1 (0.1-0.2)	0.5 (0.5-0.5)	0.1 (0.1-0.2)
**PPM+Q**	0.4 (0.4-0.5)	0.1 (0.1-0.1)	0.4 (0.4-0.4)	0.5 (0.5-0.5)	0.1 (0.1-0.1)	0.1 (0.1-0.1)	0.5 (0.5-0.5)	0.1 (0.1-0.1)
**SC+CCR+PPM**	9.8 (9.3-10.3)	3.2 (3.1-3.4)	8.8 (8.3-9.2)	7.7 (7.3-8.1)	2.9 (2.8-3.0)	2.5 (2.4-2.6)	7.6 (7.3-8.0)	2.5 (2.3-2.6)
**SC+CCR+VI**	1.2 (1.1-1.2)	0.4 (0.4-0.4)	1.0 (1.0-1.1)	1.2 (1.1-1.2)	0.3 (0.3-0.4)	0.4 (0.4-0.4)	1.2 (1.1-1.2)	0.4 (0.4-0.4)
**SC+CCR+Q**	1.1 (1.1-1.2)	0.4 (0.4-0.4)	1.0 (1.0-1.1)	1.2 (1.1-1.2)	0.3 (0.3-0.4)	0.4 (0.4-0.4)	1.2 (1.1-1.2)	0.4 (0.4-0.4)
**SC+PPM+VI**	0.8 (0.7-0.8)	0.2 (0.2-0.2)	0.7 (0.6-0.7)	0.7 (0.7-0.8)	0.2 (0.2-0.2)	0.2 (0.2-0.2)	0.7 (0.7-0.7)	0.2 (0.2-0.2)

**SC+PPM+Q**	0.7 (0.7-0.8)	0.2 (0.2-0.2)	0.7 (0.6-0.7)	0.7 (0.7-0.7)	0.2 (0.2-0.2)	0.2 (0.2-0.2)	0.7 (0.6-0.7)	0.2 (0.2-0.2)
**CCR+PPM+VI**	0.4 (0.4-0.5)	0.1 (0.1-0.1)	0.4 (0.4-0.4)	0.5 (0.5-0.5)	0.1 (0.1-0.1)	0.1 (0.1-0.1)	0.5 (0.5-0.5)	0.1 (0.1-0.1)
**CCR+PPM+Q**	0.4 (0.4-0.5)	0.1 (0.1-0.1)	0.4 (0.4-0.4)	0.5 (0.5-0.5)	0.1 (0.1-0.1)	0.1 (0.1-0.1)	0.5 (0.5-0.5)	0.1 (0.1-0.1)
**SC+CCR+PPM+VI**	0.7 (0.7-0.8)	0.2 (0.2-0.2)	0.6 (0.6-0.7)	0.7 (0.6-0.7)	0.2 (0.2-0.2)	0.2 (0.2-0.2)	0.7 (0.6-0.7)	0.2 (0.2-0.2)
**SC+CCR+PPM+Q**	0.7 (0.7-0.7)	0.2 (0.2-0.2)	0.6 (0.6-0.7)	0.7 (0.6-0.7)	0.2 (0.2-0.2)	0.2 (0.2-0.2)	0.6 (0.6-0.7)	0.2 (0.2-0.2)
