# Supplementary material for: Modelling community-control strategies to protect hospital resources during an influenza pandemic in Ottawa, Canada
Source: PLoS One. 2017 Jun 14;12(6):e0179315. doi: 10.1371/journal.pone.0179315 (PMC5470707; doi:10.1371/journal.pone.0179315)
Supplement: S5 Table — (PDF) [file pone.0179315.s006.pdf]

S5 Table. Results of Basic Analysis: Intensive Care Unit Admissions

Table S5.1 provides the best-guess results for the number of ICU admissions predicted for each of the 192 intervention bundles.

Table S5.1. Predicted number of ICU admissions (95% confidence intervals)

Non-pharmaceutical intervention component	Pharmaceutical intervention component							
	**None**	**V**	**AVT**	**AVP**	**V+AVT**	**V+AVP**	**AVT+AVP**	**V+AVT+AVP**
**None**	579.8 (578.7-580.8)	213.9 (213.5-214.2)	520.4 (519.5-521.3)	495.4 (494.5-496.2)	191.7 (191.4-192.1)	181.0 (180.7-181.3)	493.4 (492.5-494.2)	180.1 (179.8-180.4)
**SC**	570.1 (569.2-570.9)	209.5 (209.2-209.8)	511.6 (510.8-512.4)	485.9 (485.2-486.6)	187.8 (187.5-188.0)	176.8 (176.5-177.0)	483.8 (483.1-484.5)	175.8 (175.6-176.1)
**CCR**	575.8 (574.8-576.8)	211.7 (211.4-212.1)	516.8 (515.8-517.7)	490.8 (490.0-491.6)	189.8 (189.5-190.1)	178.6 (178.3-178.9)	488.7 (487.9-489.6)	177.7 (177.4-178.0)
**PPM**	464.3 (463.5-465.1)	155.3 (155.0-155.5)	415.1 (414.3-415.8)	366.5 (365.8-367.1)	138.3 (138.1-138.6)	118.8 (118.6-119.0)	362.7 (362.0-363.4)	117.2 (117.0-117.4)
**VI**	355.4 (354.9-356.0)	118.3 (118.1-118.5)	317.6 (317.2-318.1)	286.1 (285.7-286.6)	105.8 (105.7-106.0)	93.1 (93.0-93.3)	283.3 (282.9-283.7)	92.0 (91.8-92.1)
**Q**	348.1 (347.7-348.5)	115.1 (115.0-115.3)	311.0 (310.7-311.4)	279.2 (278.9-279.5)	103.0 (102.9-103.1)	90.3 (90.2-90.4)	276.3 (276.0-276.6)	89.1 (89.0-89.2)
**SC+CCR**	565.0 (564.2-565.8)	206.9 (206.6-207.1)	507.0 (506.3-507.7)	480.2 (479.5-480.9)	185.3 (185.1-185.6)	173.9 (173.7-174.2)	478.0 (477.3-478.7)	172.9 (172.7-173.2)
**SC+PPM**	446.1 (445.4-446.8)	148.0 (147.7-148.2)	398.6 (398.0-399.2)	350.4 (349.9-350.9)	131.7 (131.5-131.9)	112.8 (112.6-112.9)	346.5 (346.0-347.1)	111.3 (111.1-111.5)

**SC+VI**	322.2 (321.8-322.6)	107.2 (107.0-107.3)	287.8 (287.5-288.2)	261.6 (261.3-261.9)	95.9 (95.8-96.0)	85.4 (85.3-85.5)	258.9 (258.6-259.2)	84.3 (84.2-84.4)
**SC+Q**	315.5 (315.3-315.8)	104.4 (104.3-104.5)	281.8 (281.6-282.1)	255.4 (255.2-255.6)	93.4 (93.3-93.5)	83.0 (83.0-83.1)	252.7 (252.5-252.9)	81.9 (81.9-82.0)
**CCR+PPM**	452.0 (451.1-452.8)	149.5 (149.2-149.8)	403.8 (403.1-404.5)	353.5 (352.9-354.2)	133.1 (132.8-133.3)	113.2 (113.0-113.4)	349.6 (349.0-350.3)	111.6 (111.4-111.8)
**CCR+VI**	344.5 (344.0-345.1)	90.9 (90.8-91.1)	113.6 (113.5-113.8)	274.4 (274.0-274.8)	101.2 (101.0-101.3)	88.5 (88.4-88.6)	271.8 (271.4-272.2)	87.3 (87.2-87.4)
**CCR+Q**	340.8 (340.4-341.1)	112.0 (111.9-112.2)	304.4 (304.0-304.7)	270.8 (270.6-271.1)	99.7 (99.6-99.8)	87.1 (87.0-87.2)	268.2 (268.0-268.5)	85.9 (85.8-86.0)
**PPM+VI**	163.3 (163.0-163.6)	46.2 (46.2-46.3)	144.5 (144.3-144.8)	114.9 (114.8-115.1)	40.7 (40.7-40.8)	32.0 (31.9-32.0)	112.4 (112.3-112.6)	31.2 (31.2-31.3)
**PPM+Q**	155.7 (155.5-155.9)	43.9 (43.8-43.9)	137.7 (137.6-137.9)	109.2 (109.1-109.4)	38.6 (38.6-38.7)	30.3 (30.3-30.3)	106.8 (106.7-106.9)	29.5 (29.5-29.6)
**SC+CCR+PPM**	432.1 (431.4-432.7)	141.7 (141.5-141.9)	385.8 (385.2-386.4)	336.2 (335.7-336.7)	126.0 (125.8-126.2)	106.8 (106.7-107.0)	332.2 (331.7-332.7)	105.2 (105.1-105.4)
**SC+CCR+VI**	311.0 (310.7-311.4)	102.7 (102.6-102.8)	277.9 (277.6-278.3)	250.2 (249.9-250.5)	91.4 (91.3-91.5)	81.0 (80.9-81.1)	247.8 (247.5-248.1)	79.9 (79.8-80.0)
**SC+CCR+Q**	307.9 (307.6-308.1)	101.3 (101.2-101.4)	274.9 (274.6-275.1)	247.1 (246.8-247.3)	90.2 (90.1-90.2)	79.8 (79.7-79.8)	244.6 (244.4-244.8)	78.7 (78.6-78.8)
**SC+PPM+VI**	146.3 (146.1-146.5)	42.1 (42.0-42.1)	129.4 (129.3-129.6)	105.5 (105.3-105.6)	37.1 (37.1-37.2)	29.9 (29.8-29.9)	103.2 (103.0-103.3)	29.2 (29.1-29.2)

**SC+PPM+Q**	139.9 (139.7-140.0)	40.1 (40.0-40.1)	123.9 (123.7-124.0)	100.6 (100.5-100.7)	35.3 (35.3-35.3)	28.4 (28.4-28.4)	98.4 (98.3-98.5)	27.7 (27.7-27.7)
**CCR+PPM+VI**	150.7 (150.4-151.0)	42.1 (42.0-42.2)	133.2 (133.0-133.5)	104.9 (104.7-105.0)	37.1 (37.0-37.1)	29.0 (28.9-29.0)	101.9 (101.8-102.1)	28.3 (28.2-28.3)
**CCR+PPM+Q**	147.0 (146.8-147.2)	41.0 (40.9-41.0)	130.0 (129.8-130.1)	102.1 (102.0-102.3)	36.1 (36.0-36.1)	28.2 (28.1-28.2)	99.3 (99.2-99.4)	27.5 (27.4-27.5)
**SC+CCR+PPM+VI**	134.9 (134.7-135.1)	38.4 (38.4-38.5)	119.3 (119.1-119.4)	96.2 (96.1-96.3)	33.8 (33.8-33.8)	27.1 (27.0-27.1)	93.7 (93.6-93.8)	26.4 (26.4-26.5)
**SC+CCR+PPM+Q**	131.8 (131.7-131.9)	37.4 (37.4-37.5)	116.5 (116.4-116.6)	94.0 (93.9-94.1)	32.9 (32.9-33.0)	26.4 (26.4-26.4)	91.3 (91.3-91.4)	25.7 (25.7-25.8)
