# Supplementary material for: Modelling community-control strategies to protect hospital resources during an influenza pandemic in Ottawa, Canada
Source: PLoS One. 2017 Jun 14;12(6):e0179315. doi: 10.1371/journal.pone.0179315 (PMC5470707; doi:10.1371/journal.pone.0179315)
Supplement: S6 Table — (PDF) [file pone.0179315.s007.pdf]

S6 Table. Results of Basic Analysis: Peak ICU demand

Table S6.1 provides the best-guess results for the peak ICU bed demand (as a percentage of all ICU hospital beds in the Ottawa–Gatineau CMA) for each of the 192 intervention bundles.

Table S6.1. Predicted peak ICU bed demand (95% confidence intervals)

**Non-pharmaceutical intervention component**	**Pharmaceutical intervention component**							
	**None**	**V**	**AVT**	**AVP**	**V+AVT**	**V+AVP**	**AVT+AVP**	**V+AVT+AVP**
**None**	90.2 (85.7-94.7)	32.1 (30.5-33.7)	80.9 (76.8-84.9)	74.7 (71.0-78.4)	28.7 (27.3-30.1)	26.3 (25.0-27.6)	74.3 (70.6-78.0)	26.2 (24.9-27.5)
**SC**	87.2 (82.9-91.6)	30.9 (29.4-32.5)	78.2 (74.3-82.1)	72.2 (68.6-75.8)	27.7 (26.3-29.1)	25.4 (24.1-26.7)	71.8 (68.2-75.4)	25.2 (24.0-26.5)
**CCR**	89.0 (84.5-93.4)	31.5 (30.0-33.1)	79.8 (75.8-83.8)	73.6 (69.9-77.2)	28.2 (26.8-29.6)	25.8 (24.6-27.1)	73.1 (69.5-76.8)	25.7 (24.4-27.0)
**PPM**	70.2 (66.7-73.7)	23.6 (22.4-24.8)	62.9 (59.7-66.0)	55.7 (53.0-58.5)	21.1 (20.0-22.1)	18.4 (17.5-19.3)	55.3 (52.6-58.1)	18.2 (17.3-19.1)
**VI**	4.7 (4.5-4.9)	1.6 (1.5-1.6)	4.2 (4.0-4.4)	5.7 (5.4-6.0)	1.4 (1.3-1.5)	1.9 (1.8-2.0)	5.7 (5.4-6.0)	1.9 (1.8-2.0)
**Q**	4.6 (4.4-4.8)	1.5 (1.4-1.6)	4.1 (3.9-4.3)	5.6 (5.4-5.9)	1.4 (1.3-1.4)	1.9 (1.8-2.0)	5.6 (5.3-5.9)	1.9 (1.8-2.0)
**SC+CCR**	85.8 (81.5-90.1)	30.3 (28.8-31.8)	76.9 (73.0-80.7)	70.9 (67.3-74.4)	27.1 (25.8-28.5)	24.8 (23.6-26.1)	70.5 (66.9-74.0)	24.7 (23.4-25.9)
**SC+PPM**	67.3 (63.9-70.7)	22.5 (21.4-23.6)	60.3 (57.2-63.3)	53.4 (50.7-56.0)	20.1 (19.1-21.1)	17.5 (16.6-18.4)	52.9 (50.3-55.6)	17.4 (16.5-18.2)

**SC+VI**	8.2 (7.8-8.6)	2.7 (2.6-2.9)	7.3 (7.0-7.7)	8.3 (7.9-8.8)	2.4 (2.3-2.6)	2.8 (2.6-2.9)	8.3 (7.9-8.7)	2.7 (2.6-2.9)
**SC+Q**	8.0 (7.6-8.4)	2.7 (2.5-2.8)	7.2 (6.8-7.6)	8.2 (7.8-8.6)	2.4 (2.3-2.5)	2.7 (2.6-2.8)	8.1 (7.7-8.5)	2.7 (2.5-2.8)
**CCR+PPM**	69.0 (65.5-72.4)	23.1 (21.9-24.2)	61.8 (58.7-64.9)	54.6 (51.8-57.3)	20.6 (19.6-21.6)	17.9 (17.0-18.8)	54.2 (51.5-56.9)	17.7 (16.8-18.6)
**CCR+VI**	4.6 (4.3-4.8)	1.5 (1.4-1.6)	1.5 (1.4-1.6)	5.6 (5.3-5.9)	1.4 (1.3-1.4)	1.9 (1.8-1.9)	5.6 (5.3-5.9)	1.8 (1.8-1.9)
**CCR+Q**	4.5 (4.3-4.8)	1.5 (1.4-1.6)	4.1 (3.9-4.3)	5.6 (5.3-5.8)	1.3 (1.3-1.4)	1.8 (1.7-1.9)	5.5 (5.3-5.8)	1.8 (1.7-1.9)
**PPM+VI**	3.0 (2.9-3.2)	0.9 (0.8-0.9)	2.7 (2.6-2.8)	3.3 (3.2-3.5)	0.8 (0.7-0.8)	0.9 (0.9-0.9)	3.3 (3.1-3.4)	0.9 (0.8-0.9)
**PPM+Q**	2.9 (2.8-3.1)	0.8 (0.8-0.9)	2.6 (2.5-2.7)	3.2 (3.0-3.3)	0.7 (0.7-0.8)	0.8 (0.8-0.9)	3.1 (3.0-3.3)	0.8 (0.8-0.9)
**SC+CCR+PPM**	65.9 (62.6-69.2)	21.9 (20.8-23.0)	59.0 (56.0-61.9)	52.0 (49.4-54.6)	19.6 (18.6-20.6)	17.0 (16.1-17.8)	51.6 (49.0-54.2)	16.8 (16.0-17.7)
**SC+CCR+VI**	7.9 (7.5-8.3)	2.6 (2.5-2.8)	7.1 (6.8-7.5)	8.1 (7.7-8.5)	2.3 (2.2-2.5)	2.7 (2.5-2.8)	2.0 (7.6-8.4)	2.6 (2.5-2.8)
**SC+CCR+Q**	7.9 (7.5-8.3)	2.6 (2.5-2.7)	7.0 (6.7-7.4)	8.0 (7.6-8.4)	43.9 (40.8-47.1)	2.6 (2.5-2.8)	8.0 (7.6-8.4)	2.6 (2.5-2.7)
**SC+PPM+VI**	5.0 (4.7-5.2)	1.4 (1.3-1.5)	4.4 (4.2-4.6)	4.6 (4.3-4.8)	1.2 (1.2-1.3)	1.2 (1.2-1.3)	4.5 (4.2-4.7)	1.2 (1.2-1.3)

**SC+PPM+Q**	4.8 (4.5-5.0)	1.3 (1.3-1.4)	4.3 (4.0-4.5)	4.4 (4.1-4.6)	1.2 (1.1-1.2)	1.2 (1.1-1.2)	4.3 (4.1-4.5)	1.2 (1.1-1.2)
**CCR+PPM+VI**	2.9 (2.7-3.0)	0.8 (0.7-0.8)	2.6 (2.4-2.7)	3.1 (2.9-3.2)	0.7 (0.7-0.7)	0.8 (0.8-0.9)	3.0 (2.9-3.2)	0.8 (0.8-0.8)
**CCR+PPM+Q**	2.8 (2.7-3.0)	0.8 (0.7-0.8)	2.5 (2.4-2.6)	3.0 (2.8-3.1)	0.7 (0.6-0.7)	0.8 (0.8-0.8)	2.9 (2.8-3.1)	0.8 (0.7-0.8)
**SC+CCR+PPM+VI**	4.6 (4.4-4.9)	1.3 (1.2-1.4)	4.1 (3.9-4.3)	4.2 (4.0-4.4)	1.1 (1.1-1.2)	1.1 (1.1-1.2)	4.1 (3.9-4.3)	1.1 (1.1-1.2)
**SC+CCR+PPM+Q**	4.5 (4.3-4.8)	1.3 (1.2-1.3)	4.0 (3.8-4.2)	4.1 (3.9-4.3)	1.1 (1.1-1.2)	1.1 (1.0-1.2)	4.0 (3.8-4.2)	1.1 (1.0-1.1)
