# Supplementary material for: Modelling community-control strategies to protect hospital resources during an influenza pandemic in Ottawa, Canada
Source: PLoS One. 2017 Jun 14;12(6):e0179315. doi: 10.1371/journal.pone.0179315 (PMC5470707; doi:10.1371/journal.pone.0179315)
Supplement: S7 Table — (PDF) [file pone.0179315.s008.pdf]

S7 Table. Results of Basic Analysis: Mortality

Table S7.1 provides the best-guess results for the number of deaths predicted for each of the 192 intervention bundles.

Table S7.1. Predicted number of deaths (95% confidence intervals)

Non-pharmaceutical intervention component	Pharmaceutical intervention component							
	None	V	AVT	AVP	V+AVT	V+AVP	AVT+AVP	V+AVT+AVP
**None**	363.0 (339.6-386.5)	131.7 (123.3-140.1)	325.6 (304.6-346.6)	305.6 (286.0-325.1)	117.9 (110.4-125.5)	109.6 (102.6-116.5)	303.9 (284.5-323.4)	108.8 (101.9-115.8)
**SC**	355.3 (333.1-377.6)	128.3 (120.4-136.3)	318.6 (298.6-338.5)	298.2 (279.6-316.7)	114.8 (107.7-122.0)	106.4 (99.8-113.0)	296.4 (278.0-314.9)	105.6 (99.1-112.2)
**CCR**	358.3 (335.2-381.5)	129.5 (121.2-137.8)	321.3 (300.5-342.1)	300.6 (281.3-319.9)	115.9 (108.4-123.3)	107.2 (100.4-114.1)	298.9 (279.7-318.1)	106.5 (99.6-113.3)
**PPM**	249.9 (230.7-269.1)	81.0 (74.6-87.5)	222.9 (205.7-240.1)	191.6 (176.4-206.9)	72.0 (66.2-77.7)	60.5 (55.5-65.4)	189.1 (174.0-204.3)	59.5 (54.6-64.4)
**VI**	184.5 (170.8-198.2)	59.6 (55.0-64.1)	164.5 (152.2-176.8)	145.3 (134.3-156.3)	53.3 (49.2-57.4)	46.1 (42.5-49.6)	143.5 (140.0-146.9)	45.4 (41.8-48.9)
**Q**	179.2 (165.8-192.7)	57.5 (53.1-62.0)	159.8 (147.7-171.8)	140.7 (129.9-151.5)	51.5 (47.5-55.4)	44.3 (40.9-47.8)	138.8 (128.1-149.5)	43.7 (40.2-47.1)
**SC+CCR**	349.8 (327.9-371.7)	125.7 (117.9-133.5)	313.5 (293.9-333.2)	292.3 (274.0-310.5)	112.4 (105.4-119.4)	103.7 (97.2-110.1)	290.5 (272.3-308.7)	102.9 (96.5-109.3)
**SC+PPM**	238.0 (220.0-255.9)	76.6 (70.6-82.6)	212.1 (196.0-228.2)	181.8 (167.6-195.9)	68.0 (62.7-73.3)	57.0 (52.5-61.6)	179.3 (165.3-193.3)	56.2 (51.7-60.7)

**SC+VI**	165.0 (153.6-176.4)	53.2 (49.5-57.0)	147.1 (136.9-157.3)	131.2 (121.9-140.4)	47.6 (44.3-51.0)	41.7 (38.7-44.6)	129.4 (120.3-138.6)	41.0 (38.1-44.0)
**SC+Q**	160.3 (149.1-171.5)	51.5 (47.8-55.1)	142.9 (132.9-152.9)	127.1 (118.0-136.1)	46.0 (42.8-49.3)	40.2 (37.3-43.1)	125.4 (116.4-134.3)	39.6 (36.8-42.4)
**CCR+PPM**	240.1 (221.2-258.9)	77.0 (70.7-83.3)	214.0 (197.1-230.9)	182.4 (167.6-197.3)	68.4 (62.8-74.0)	56.9 (52.1-61.7)	179.9 (165.2-194.6)	56.0 (51.2-60.7)
**CCR+VI**	176.6 (163.2-189.9)	56.5 (52.2-60.8)	56.5 (52.2-60.9)	137.3 (126.6-148.0)	50.2 (46.3-54.1)	43.2 (39.8-46.6)	135.8 (125.2-146.3)	42.5 (39.1-45.9)
**CCR+Q**	173.9 (160.7-187.1)	55.5 (51.2-59.9)	155.0 (143.2-166.8)	135.0 (124.4-145.5)	49.3 (45.5-53.2)	42.3 (39.0-45.7)	133.4 (123.0-143.9)	41.7 (38.3-45.0)
**PPM+VI**	71.2 (64.1-78.3)	20.2 (18.3-22.2)	62.9 (56.6-69.2)	50.2 (45.3-55.1)	17.8 (16.1-19.5)	14.1 (12.8-15.4)	49.0 (4.2-53.8)	13.8 (12.5-15.0)
**PPM+Q**	67.6 (60.8-74.4)	19.1 (17.3-21.0)	59.7 (53.7-65.7)	47.5 (42.9-52.2)	16.8 (15.2-18.4)	13.4 (12.1-14.6)	46.4 (41.9-51.0)	13.0 (11.8-14.2)
**SC+CCR+PPM**	227.4 (209.9-244.9)	72.4 (66.7-78.2)	202.5 (186.9-218.2)	172.2 (158.5-185.8)	64.2 (59.1-69.4)	53.4 (49.1-57.8)	169.6 (156.1-183.2)	52.5 (48.2-56.8)
**SC+CCR+VI**	157.2 (146.2-168.3)	50.4 (46.8-54.0)	140.2 (130.4-150.1)	123.6 (114.7-132.5)	44.7 (41.5-47.9)	39.0 (36.2-41.8)	122.1 (113.3-130.9)	38.4 (35.6-41.1)
**SC+CCR+Q**	155.1 (144.1-166.0)	49.5 (46.0-53.0)	138.1 (128.4-147.9)	121.5 (112.8-130.3)	43.9 (40.8-47.1)	38.2 (35.5-41.0)	120.1 (111.4-128.8)	37.6 (34.9-40.4)
**SC+PPM+VI**	62.8 (57.2-68.3)	18.1 (16.6-19.6)	55.4 (50.6-60.3)	45.3 (41.4-49.2)	16.0 (14.6-17.3)	13.0 (12.0-14.1)	44.3 (40.5-48.1)	12.7 (11.7-13.7)

**SC+PPM+Q**	59.7 (54.4-64.9)	17.2 (15.8-18.7)	52.8 (48.2-57.5)	43.1 (39.4-46.9)	15.1 (13.9-16.4)	12.4 (11.4-13.4)	42.1 (38.5-45.8)	12.1 (11.1-13.0)
**CCR+PPM+VI**	65.1 (58.5-71.6)	18.3 (16.5-20.0)	57.5 (51.6-63.3)	45.4 (40.9-49.9)	16.1 (14.5-17.6)	12.7 (1.6-13.9)	44.1 (39.8-48.5)	12.4 (11.3-13.6)
**CCR+PPM+Q**	63.3 (56.9-69.7)	17.8 (16.0-19.5)	55.9 (50.3-61.6)	44.2 (39.8-48.5)	15.6 (14.1-17.1)	12.4 (11.2-13.5)	42.9 (38.7-47.1)	12.1 (11.0-13.2)
**SC+CCR+PPM+VI**	57.3 (52.2-62.4)	16.4 (15.1-17.8)	50.6 (46.1-55.1)	41.0 (37.5-44.6)	14.5 (12.2-15.7)	11.8 (10.8-12.7)	40.0 (36.5-43.4)	11.5 (10.6-12.4)
**SC+CCR+PPM+Q**	55.9 (50.9-60.8)	16.0 (14.6-17.3)	49.3 (44.9-53.7)	40.1 (36.6-43.6)	14.1 (12.9-15.3)	11.5 (10.5-12.4)	38.9 (35.5-42.3)	11.2 (10.3-12.1)
