# Supplementary material for: Modelling community-control strategies to protect hospital resources during an influenza pandemic in Ottawa, Canada
Source: PLoS One. 2017 Jun 14;12(6):e0179315. doi: 10.1371/journal.pone.0179315 (PMC5470707; doi:10.1371/journal.pone.0179315)
Supplement: S8 Table — (PDF) [file pone.0179315.s009.pdf]

# S8 Table. Economic Analyses for Seven Intervention Bundles of Interest

**Tables S8.1–S8.7** present the cost-breakdown for the seven key intervention bundles of interest. Intervention bundles involving either no interventions or pharmaceutical measures only produced estimates of economic burden dominated by mortality and healthcare utilization. Meanwhile, lost school and work days were responsible for much of the economic burden associated with non-pharmaceutical measures such as voluntary isolation and school closure.

**Table S8.1**. **Economic analysis for "No Intervention" scenario**

| Category | Total | Unit Cost | Cost |
|---|---|---|---|
| Total hospital bed days | 2,472 | 1,042 | 10,303,296 |
| Total ICU bed days | 580 | 2,084 | 12,087,200 |
| Total deaths (Infant) | 4 | 2,355,172 | 9,420,688 |
| Total deaths (child) | 4 | 2,207,744 | 8,830,976 |
| Total deaths (young adult) | 27 | 1,956,694 | 52,830,738 |
| Total deaths (adult) | 179 | 1,374,086 | 245,961,394 |
| Total deaths (seniors) | 150 | 424,296 | 63,644,400 |
| Total lost school days | 786 | 92 | 72,194 |
| Total lost work days (YA + Adults) | 9,448 | 193 | 1,819,212 |
| Total lost work days (Senior) | 435 | 193 | 83,759 |
| Total vaccinations | 0 | 20 | 0 |
| Total antivirals | 0 | 25 | 0 |
| Total masks | 0 | 4 | 0 |
| Total | | | 405,053,858 |

**Table S8.2. Economic analysis for "Vaccination and Antiviral Therapy" scenario**

| Category | Total | Unit Cost | Cost |
|---|---|---|---|
| Total hospital bed days | 815 | 1,042 | 3,396,920 |
| Total ICU bed days | 192 | 2,084 | 4,001,280 |
| Total deaths (Infant) | 1 | 2,355,172 | 2,355,172 |
| Total deaths (child) | 1 | 2,207,744 | 2,207,744 |
| Total deaths (young adult) | 9 | 1,956,694 | 17,610,246 |
| Total deaths (adult) | 58 | 1,374,086 | 79,696,988 |
| Total deaths (seniors) | 49 | 424,296 | 20,790,504 |
| Total lost school days | 256 | 92 | 23,514 |
| Total lost work days (YA + Adults) | 3,118 | 193 | 600,371 |
| Total lost work days (Senior) | 133 | 193 | 25,609 |
| Total vaccinations | 436,212 | 20 | 8,724,240 |
| Total antivirals | 311,341 | 25 | 7,783,530 |
| Total masks | 0 | 4 | 0 |
| Total | | | 147,216,118 |

**Table S8.3. Economic analysis for "Vaccination, Antiviral Therapy, and Antiviral Prophylaxis" scenario**

| Category | Total | Unit Cost | Cost |
|---|---|---|---|
| Total hospital bed days | 765 | 1,042 | 3,188,520 |
| Total ICU bed days | 180 | 2,084 | 3,751,200 |
| Total deaths (Infant) | 1 | 2,355,172 | 2,355,172 |
| Total deaths (child) | 1 | 2,207,744 | 2,207,744 |
| Total deaths (young adult) | 8 | 1,956,694 | 15,653,552 |
| Total deaths (adult) | 54 | 1,374,086 | 74,200,644 |
| Total deaths (seniors) | 45 | 424,296 | 19,093,320 |
| Total lost school days | 238 | 92 | 21,860 |
| Total lost work days (YA + Adults) | 2,942 | 193 | 566,482 |
| Total lost work days (Senior) | 124 | 193 | 23,876 |
| Total vaccinations | 436,212 | 20 | 8,724,240 |
| Total antivirals | 361,935 | 25 | 9,048,375 |
| Total masks | 0 | 4 | 0 |
| Total | | | 138,834,986 |

## Table S8.4. Economic analysis for "Community-Contact Reduction, Personal Protective Measures, and Voluntary Isolation" scenario

| Category | Total | Unit Cost | Cost |
|---|---|---|---|
| Total hospital bed days | 634 | 1,042 | 2,642,512 |
| Total ICU bed days | 151 | 2,084 | 3,146,840 |
| Total deaths (Infant) | 1 | 2,355,172 | 2,355,172 |
| Total deaths (child) | 1 | 2,207,744 | 2,207,744 |
| Total deaths (young adult) | 4 | 1,956,694 | 7,826,776 |
| Total deaths (adult) | 35 | 1,374,086 | 48,093,010 |
| Total deaths (seniors) | 25 | 424,296 | 10,607,400 |
| Total lost school days | 54,921 | 92 | 5,044,494 |
| Total lost work days (YA + Adults) | 197,799 | 193 | 38,086,197 |
| Total lost work days (Senior) | 3,032 | 193 | 583,812 |
| Total vaccinations | 0 | 20 | 0 |
| Total antivirals | 0 | 25 | 0 |
| Total masks | 186,948 | 4 | 747,792 |
| Total | | | 121,341,749 |

## Table S8.5. Economic analysis for "Community-Contact Reduction, Personal Protective Measures, Voluntary Isolation and Antiviral Therapy" scenario

| Category | Total | Unit Cost | Cost |
|---|---|---|---|
| Total hospital bed days | 560 | 1,042 | 2,334,080 |
| Total ICU bed days | 133 | 2,084 | 2,771,720 |
| Total deaths (Infant) | 1 | 2,355,172 | 2,355,172 |
| Total deaths (child) | 1 | 2,207,744 | 2,207,744 |
| Total deaths (young adult) | 4 | 1,956,694 | 7,826,776 |
| Total deaths (adult) | 31 | 1,374,086 | 42,596,666 |
| Total deaths (seniors) | 22 | 424,296 | 9,334,512 |
| Total lost school days | 54,024 | 92 | 4,962,104 |
| Total lost work days (YA + Adults) | 194,391 | 193 | 37,429,987 |
| Total lost work days (Senior) | 2,972 | 193 | 572,259 |
| Total vaccinations | 0 | 20 | 0 |
| Total antivirals | 100,264 | 25 | 2,506,608 |
| Total masks | 186,948 | 4 | 747,792 |
| Total | | | 115,645,420 |

**Table S8.6 Economic analysis for "School Closure, Community Contact Reduction, Personal Protective Measures, Voluntary Isolation and Quarantine" scenario**

| Category | Total | Unit Cost | Cost |
|---|---|---|---|
| Total hospital bed days | 550 | 1,042 | 2,292,400 |
| Total ICU bed days | 132 | 2,084 | 2,750,880 |
| Total deaths (Infant) | 1 | 2,355,172 | 2,355,172 |
| Total deaths (child) | 1 | 2,207,744 | 2,207,744 |
| Total deaths (young adult) | 4 | 1,956,694 | 7,826,776 |
| Total deaths (adult) | 24 | 1,374,086 | 32,978,064 |
| Total deaths (seniors) | 25 | 424,296 | 10,607,400 |
| Total lost school days | 9,916,572 | 92 | 910,837,138 |
| Total lost work days (YA + Adults) | 6,125,219 | 193 | 1,179,410,918 |
| Total lost work days (Senior) | 3,114 | 193 | 599,601 |
| Total vaccinations | 0 | 20 | 0 |
| Total antivirals | 0 | 25 | 0 |
| Total masks | 186,948 | 4 | 747,792 |
| Total | | | 2,152,613,885 |

**Table S8.7. Economic analysis for "School Closure, Community Contact Reduction, Personal Protective Measures, Voluntary Isolation, Quarantine, Vaccination, Antiviral Therapy and Antiviral Prophylaxis" scenario**

| Category | Total | Unit Cost | Cost |
|---|---|---|---|
| Total hospital bed days | 108 | 1,042 | 450,144 |
| Total ICU bed days | 26 | 2,084 | 541,840 |
| Total deaths (Infant) | 0 | 2,355,172 | 0 |
| Total deaths (child) | 0 | 2,207,744 | 0 |
| Total deaths (young adult) | 1 | 1,956,694 | 1,956,694 |
| Total deaths (adult) | 5 | 1,374,086 | 6,870,430 |
| Total deaths (seniors) | 5 | 424,296 | 2,121,480 |
| Total lost school days | 8,649,572 | 92 | 794,463,188 |
| Total lost work days (YA + Adults) | 5,313,083 | 193 | 1,023,034,132 |
| Total lost work days (Senior) | 1,632 | 193 | 314,242 |
| Total vaccinations | 436,212 | 20 | 8,724,240 |
| Total antivirals | 103,725 | 25 | 2,593,125 |
| Total masks | 186,948 | 4 | 747,792 |
| Total | | | 1,841,817,306 |
