# Supplementary material for: Modelling community-control strategies to protect hospital resources during an influenza pandemic in Ottawa, Canada
Source: PLoS One. 2017 Jun 14;12(6):e0179315. doi: 10.1371/journal.pone.0179315 (PMC5470707; doi:10.1371/journal.pone.0179315)
Supplement: S9 Table — (PDF) [file pone.0179315.s010.pdf]

S9 Table. Results of Sensitivity Analysis

Tables S9.1–S9.4 present outcome measure counts for best-guess (BG), worst-case (WC), and best-case (BC) scenarios for each individual intervention. Below each WC and BC scenario is a measure of the percent change in each outcome measure, relative to the BG scenario. Sensitivity analysis were conducted across four pandemic scenarios where transmissibility was calculated to approximate an R_o of either 1.65 (1957 pandemic) or 1.80 (1918 pandemic) and the hospitalization rate was calibrated to approximate a rate of either 0.4% or 1.0%

Table S9.1. Sensitivity of health outcome predictions to change in intervention parameter assumptions for a pandemic strain with the transmissibility of the 1957 pandemic and a hospitalization rate of 0.4%

Intervention	Measure	Outcome					
		Symptomatic Cases	Hospitalizations	ICU	Peak Hospital Demand	Peak ICU Demand	Deaths
No Intervention	Count	677,545.6	2,472.0	579.8	13.8	90.2	363.0
SC — BG	Count	669,919.6	2,430.1	570.1	13.3	87.2	355.3
SC — WC	Count	682,470.7	2,480.2	581.8	14.0	91.3	364.9
	Percent change	1.9%	2.1%	2.0%	5.0%	4.6%	2.7%
SC — BC	Count	643,517.9	2,321.0	544.9	12.1	79.9	334.9
	Percent change	-3.9%	-4.5%	-4.4%	-9.0%	-8.4%	-5.7%
CCR — BG	Count	673,282.2	2,452.3	575.8	13.6	89.0	358.3
CCR — WC	Count	675,443.6	2,462.3	577.8	13.7	89.6	360.7
	Percent change	0.3%	0.4%	0.4%	0.8%	0.7%	0.7%
CCR — BC	Count	671,058.4	2,442.0	573.7	13.5	88.4	355.8
	Percent change	-0.6%	-0.8%	-0.7%	-1.6%	-1.3%	-1.4%
PPM — BG	Count	566,467.1	1,956.7	464.3	10.5	70.2	249.9
PPM — WC	Count	673,750.5	2,454.6	576.2	13.6	89.1	358.9
	Percent change	18.9%	25.4%	24.1%	29.7%	26.9%	43.6%
PPM — BC	Count	106,153.1	3,12.3	71.3	2.8	15.4	30.9
	Percent change	-81.3%	-84.0%	-84.6%	-73.5%	-78.0%	-87.6%
VI — BG	Count	429,992.9	1,479.9	355.4	0.7	4.7	184.5
VI — WC	Count	508,807.0	1,789.6	426.6	2.3	15.6	236.4
	Percent change	18.3%	20.9%	20.0%	237.6%	233.5%	28.1%

VI — BC	Count	407,053.3	1,391.4	335.1	0.4	2.5	170.1
	Percent change	-20.0%	-22.2%	-21.4%	-84.5%	-84.3%	-28.0%
Q — BG	Count	422,402.4	1,448.9	348.1	0.7	4.6	179.2
Q — WC	Count	503,849.9	1,768.5	421.8	2.3	15.4	232.4
	Percent change	19.3%	22.1%	21.2%	239.4%	235.3%	29.7%
Q — BC	Count	398,705.2	1,357.8	327.2	0.4	2.4	164.6
	Percent change	-5.6%	-6.3%	-6.0%	-47.8%	-47.7%	-8.1%
V — BG	Count	624,359.9	909.7	213.9	4.9	32.1	131.7
V — WC	Count	667,013.6	1,827.8	428.7	10.1	66.4	268.4
	Percent change	6.8%	100.9%	100.4%	108.4%	107.2%	103.8%
V — BC	Count	4,375.1	1.4	0.3	0.0	0.0	0.2
	Percent change	-99.3%	-99.8%	-99.8%	-99.9%	-99.9%	-99.9%
AVT — BG	Count	676,261.7	2,218.6	520.4	12.4	80.9	325.6
AVT — WC	Count	676,838.9	2,468.1	578.9	13.8	90.0	362.3
	Percent change	0.1%	11.2%	11.2%	11.3%	11.3%	11.3%
AVT — BC	Count	676,720.4	1,481.7	347.5	8.3	54.0	217.6
	Percent change	0.0%	0.0%	0.1%	5.4%	0.8%	0.2%
AVP — BG	Count	658,479.8	2,110.8	495.4	11.4	74.7	305.6
AVP — WC	Count	658,842.0	2,422.4	568.0	13.3	87.3	353.6
	Percent change	0.1%	14.8%	14.7%	17.3%	16.8%	15.7%
AVP — BC	Count	673,938.2	1,385.8	325.0	7.4	48.8	200.3
	Percent change	2.3%	-34.3%	-34.4%	-34.7%	-34.6%	-34.5%

Table S9.2. Sensitivity of health outcome predictions to change in intervention parameter assumptions for a pandemic strain with the transmissibility of the 1918 pandemic and a hospitalization rate of 0.4%

Intervention	Measure	Outcome					
		Symptomatic Cases	Hospitalizations	ICU	Peak Hospital Demand	Peak ICU Demand	Deaths
No Intervention	Count	713,919.5	2,632.9	612.4	16.0	103.1	400.2
SC — BG	Count	708,301.8	2,603.5	605.4	15.5	100.1	395.2
SC — WC	Count	717,502.1	2,637.2	613.4	16.2	104.0	401.1
	Percent change	1.3%	1.3%	1.3%	4.4%	3.9%	1.5%
SC — BC	Count	688,700.8	2,526.6	587.7	14.3	92.8	381.3
	Percent change	-2.8%	-3.0%	-2.9%	-8.0%	-7.2%	-3.5%
CCR — BG	Count	711,061.4	2,620.7	609.9	15.8	101.8	397.4
CCR — WC	Count	712,504.5	2,626.9	611.1	15.9	102.5	398.8
	Percent change	0.2%	0.2%	0.2%	0.6%	0.7%	0.3%
CCR — BC	Count	709,592.0	2,596.2	391.7	15.6	73.0	605.0
	Percent change	-0.4%	-1.2%	-35.9%	-1.9%	-28.8%	51.7%
PPM — BG	Count	651,004.0	2,348.7	553.9	12.7	83.4	333.9
PPM — WC	Count	711,370.9	2,622.0	610.1	15.8	102.0	397.7
	Percent change	9.3%	11.6%	10.2%	25.1%	22.3%	19.1%
PPM — BC	Count	281,302.8	858.7	198.5	6.9	41.5	88.2
	Percent change	-56.8%	-63.4%	-64.2%	-45.7%	-50.3%	-73.6%
VI — BG	Count	517,475.0	1,855.1	442.1	0.9	5.8	255.9
VI — WC	Count	581,944.8	2,112.3	498.9	2.9	19.0	303.2
	Percent change	12.5%	13.9%	12.8%	232.9%	228.8%	18.5%
VI — BC	Count	498,423.1	1,779.5	425.4	0.5	3.0	242.1
	Percent change	-14.4%	-15.8%	-14.7%	-84.3%	-84.0%	-20.1%
Q — BG	Count	512,432.0	1,833.7	437.4	0.9	5.7	251.6
Q — WC	Count	578,733.5	2,098.7	496.0	2.9	18.8	300.4
	Percent change	12.9%	14.5%	13.4%	234.7%	230.4%	19.4%
Q — BC	Count	492,790.4	1,755.7	420.1	0.4	3.0	237.5
	Percent change	-3.8%	-4.3%	-3.9%	-47.7%	-47.6%	-5.6%

V — BG	Count	670,681.0	991.2	230.9	5.8	37.5	149.8
V — WC	Count	706,942.8	1,958.6	455.5	11.9	76.5	297.7
	Percent change	5.4%	97.6%	97.3%	105.4%	103.9%	98.8%
V — BC	Count	13,155.2	4.1	0.9	0.0	0.2	0.4
	Percent change	-98.0%	-99.6%	-99.6%	-99.5%	-99.6%	-99.7%
AVT — BG	Count	712,964.6	2,364.7	550.0	14.4	92.5	359.3
AVT — WC	Count	713,372.2	2,629.7	611.7	16.0	102.9	399.6
	Percent change	0.1%	11.2%	11.2%	11.3%	11.3%	11.2%
AVT — BC	Count	713,307.3	1,579.0	367.2	9.6	61.8	240.0
	Percent change	0.0%	0.0%	0.1%	4.7%	0.7%	0.2%
AVP — BG	Count	702,167.6	2,285.8	531.6	13.4	87.0	345.1
AVP — WC	Count	698,623.8	2,596.4	603.6	15.6	100.5	393.4
	Percent change	-0.5%	13.6%	13.5%	16.1%	15.5%	14.0%
AVP — BC	Count	717,796.4	1,505.3	349.9	8.8	57.0	227.0
	Percent change	2.2%	-34.1%	-34.2%	-34.5%	-34.5%	-34.2%

Table S9.3. Sensitivity of health outcome predictions to change in intervention parameter assumptions for a pandemic strain with the transmissibility of the 1957 pandemic and hospitalization rate of 1.0%

Intervention	Measure	Outcome					
		Symptomatic Cases	Hospitalizations	ICU	Peak Hospital Demand	Peak ICU Demand	Deaths
No Intervention	Count	675,698.9	4,893.4	1,148.8	27.3	178.5	717.1
SC — BG	Count	667,946.9	4,808.6	1,129.2	26.3	172.5	701.5
SC — WC	Count	680,669.8	4,909.8	1,152.8	27.6	180.6	720.8
	Percent change	1.9%	2.1%	2.1%	5.1%	4.7%	2.8%
SC — BC	Count	641,209.9	4,589.0	1,078.4	23.9	157.9	660.4
	Percent change	-4.0%	-4.6%	-4.5%	-9.0%	-8.5%	-5.9%
CCR — BG	Count	671,368.1	4,853.8	1,140.7	26.9	176.0	707.6
CCR — WC	Count	673,563.9	4,873.9	1,144.8	27.1	177.1	712.5
	Percent change	0.3%	0.4%	0.4%	0.8%	0.6%	0.7%
CCR — BC	Count	669,108.4	4,833.1	1,136.4	26.6	174.8	702.7
	Percent change	-0.7%	-0.8%	-0.7%	-1.6%	-1.3%	-1.4%
PPM — BG	Count	562,395.5	3,853.2	914.8	20.7	138.7	490.0
PPM — WC	Count	671,844.6	4,858.4	1,141.5	26.9	176.3	708.8
	Percent change	19.5%	26.1%	24.8%	29.9%	27.1%	44.7%
PPM — BC	Count	102,187.0	597.9	136.5	5.3	29.5	59.2
	Percent change	-81.8%	-84.5%	-85.1%	-74.3%	-78.7%	-87.9%
VI — BG	Count	426,327.9	2,910.6	699.5	1.4	9.3	361.2
VI — WC	Count	505,743.8	3,529.0	842.0	4.6	31.0	464.4
	Percent change	18.6%	21.2%	20.4%	237.5%	233.5%	28.6%
VI — BC	Count	403,230.5	2,734.3	659.0	0.7	4.9	332.8
	Percent change	-20.3%	-22.5%	-21.7%	-84.5%	-84.3%	-28.3%
Q — BG	Count	418,648.4	2,848.6	684.8	1.3	9.1	350.8
Q — WC	Count	500,721.9	3,486.8	832.2	4.6	30.6	456.5
	Percent change	19.6%	22.4%	21.5%	239.3%	235.4%	30.1%
Q — BC	Count	394,791.5	2,667.0	643.0	0.7	4.8	321.9
	Percent change	-5.7%	-6.4%	-6.1%	-47.8%	-47.7%	-8.2%

V — BG	Count	623,468.2	1,811.3	426.0	9.7	63.8	262.0
V — WC	Count	665,541.0	3,626.8	851.2	20.1	131.7	531.8
	Percent change	6.7%	100.2%	99.8%	107.5%	106.4%	103.0%
V — BC	Count	4,357.5	2.8	0.7	0.0	0.1	0.3
	Percent change	-99.3%	-99.8%	-99.8%	-99.8%	-99.9%	-99.9%
AVT — BG	Count	674,591.3	4,396.2	1,032.1	24.5	160.2	643.9
AVT — WC	Count	674,988.5	4,885.7	1,147.1	27.2	178.1	715.7
	Percent change	0.1%	11.1%	11.1%	11.2%	11.2%	11.2%
AVT — BC	Count	675,608.4	2,945.2	691.0	16.4	107.3	431.9
	Percent change	0.0%	0.0%	0.1%	2.7%	0.4%	0.1%
AVP — BG	Count	656,540.0	4,180.5	981.9	22.5	147.9	603.9
AVP — WC	Count	656,880.9	4,794.2	1,125.2	26.3	172.6	698.3
	Percent change	0.1%	14.7%	14.6%	17.1%	16.7%	15.6%
AVP — BC	Count	672,615.9	2,753.5	646.1	14.7	97.0	397.3
	Percent change	2.4%	-34.1%	-34.2%	-34.5%	-34.4%	-34.2%

Table S9.4. Sensitivity of health outcome predictions to change in intervention parameter assumptions for a pandemic strain with the transmissibility of the 1918 pandemic and hospitalization rate of 1.0%

Intervention	Measure	Outcome					
		Symptomatic Cases	Hospitalizations	ICU	Peak Hospital Demand	Peak ICU Demand	Deaths
No Intervention	Count	712,553.4	5,216.9	1,214.4	31.7	204.2	791.7
SC — BG	Count	706,873.6	5,157.4	1,200.3	30.7	198.1	781.7
SC — WC	Count	716,149.3	5,225.3	1,216.5	32.0	206.1	793.6
	Percent change	1.3%	1.3%	1.3%	4.4%	4.0%	1.5%
SC — BC	Count	687,160.4	5,003.9	1,165.0	28.2	183.8	754.1
	Percent change	-2.8%	-3.0%	-2.9%	-8.0%	-7.2%	-3.5%
CCR — BG	Count	709,661.9	5,192.2	1,209.4	31.3	201.6	786.2
CCR — WC	Count	711,121.9	5,204.7	1,211.9	31.5	202.9	789.0
	Percent change	0.2%	0.2%	0.2%	0.7%	0.6%	0.4%
CCR — BC	Count	708,172.2	5,179.5	1,206.9	31.0	200.6	783.3
	Percent change	-0.4%	-0.5%	-0.4%	-1.4%	-1.2%	-0.7%
PPM — BG	Count	648,707.8	4,644.8	1,096.4	25.0	164.9	658.5
PPM — WC	Count	709,975.7	5,195.0	1,209.9	31.3	201.9	786.9
	Percent change	9.4%	11.8%	10.4%	25.2%	22.4%	19.5%
PPM — BC	Count	274,245.3	1,661.5	384.1	13.4	80.5	170.2
	Percent change	-57.7%	-64.2%	-65.0%	-46.5%	-51.2%	-74.2%
VI — BG	Count	515,054.4	3,664.3	874.1	1.7	11.5	503.9
VI — WC	Count	579,966.5	4,178.2	987.8	5.7	37.8	598.3
	Percent change	12.6%	14.0%	13.0%	232.7%	228.9%	18.7%
VI — BC	Count	495,861.7	3,513.3	840.7	0.9	6.0	476.6
	Percent change	-14.5%	-15.9%	-14.9%	-84.2%	-84.0%	-20.4%
Q — BG	Count	509,956.5	3,621.5	864.6	1.7	11.3	495.5
Q — WC	Count	576,724.8	4,151.0	981.9	5.7	37.4	592.8
	Percent change	13.1%	14.6%	13.6%	235.0%	230.1%	19.6%
Q — BC	Count	490,166.7	3,465.6	830.0	0.9	5.9	467.3
	Percent change	-3.9%	-4.3%	-4.0%	-47.6%	-47.6%	-5.7%

V — BG	Count	670,041.2	1,974.5	460.0	11.5	74.7	298.2
V — WC	Count	705,855.8	3,889.3	905.1	23.6	152.0	590.5
	Percent change	5.3%	97.0%	96.7%	104.5%	103.3%	98.1%
V — BC	Count	13,099.8	8.1	1.9	0.1	0.3	0.9
	Percent change	-98.0%	-99.6%	-99.6%	-99.5%	-99.6%	-99.7%
AVT — BG	Count	711,731.3	4,689.8	1,091.6	28.4	183.3	711.7
AVT — WC	Count	712,004.8	5,210.5	1,213.0	31.6	203.8	790.6
	Percent change	0.0%	11.1%	11.1%	11.2%	11.2%	11.1%
AVT — BC	Count	712,485.2	3,140.2	730.6	19.0	122.8	476.9
	Percent change	0.0%	0.0%	0.1%	2.4%	0.4%	0.1%
AVP — BG	Count	700,792.0	4,532.4	1,055.0	26.6	172.3	683.2
AVP — WC	Count	697,189.4	5,144.1	1,196.9	30.8	198.9	778.2
	Percent change	-0.5%	13.5%	13.5%	16.0%	15.4%	13.9%
AVP — BC	Count	716,872.9	2,993.1	696.0	17.5	113.3	450.9
	Percent change	2.3%	-34.0%	-34.0%	-34.3%	-34.3%	-34.0%
